# Cyano-Substituted Oligo(*p*-phenylene vinylene) Derivatives with Aggregation-Induced Enhanced Emissions and Mechanofluorochromic Luminescence

**DOI:** 10.3390/molecules29184447

**Published:** 2024-09-19

**Authors:** Xinju Zhu, Yaru Pan, Xinran Zhao, Yu Yuan, Zewen Zhai, Xiaoni Yu, Wenjing Zhang, Yuanyuan Chang, Bing Song, Linlin Shi, Xinqi Hao

**Affiliations:** College of Chemistry, Zhengzhou University, No. 100 of Science Road, Zhengzhou 450001, China; pyr12042023@163.com (Y.P.); zxr6121@163.com (X.Z.); 19561343255@163.com (Y.Y.); zzwen0403@163.com (Z.Z.); yuxn0802@163.com (X.Y.); zhangwj@zzu.edu.cn (W.Z.); bingsong@zzu.edu.cn (B.S.); linlinshi@zzu.edu.cn (L.S.)

**Keywords:** CN-substituted oligo(*p*-phenylene vinylene), intramolecular charge transfer, mechanofluorochromic properties, aggregation-induced enhanced emission

## Abstract

Developing red fluorescence emitters with simple structures via convenient synthetic routes is highly desirable yet challenging. Herein, two novel donor–acceptor-type red emitters, DCFOPV-TPA and SCFOPV-TPA, featuring the intramolecular charge transfer effect were designed by integrating triphenylamine and trifluoromethyl into a CN-substituted oligo(*p*-phenylene vinylene) backbone. Both chromophores exhibited aggregation-induced enhanced emission and solvatochromic behavior. Moreover, DCFOPV-TPA also displayed reversible mechanofluorochromic properties under external force.

## 1. Introduction

Mechanofluorochromic (MFC) organic materials, which respond to external physical stimuli, such as shearing, grinding, or compressing [1,2,3], have attracted much attention due to their potential applications in memory sensors [4], security inks [5], data storage [6], fluorescence switches [7], fluorescent probes [8], and optoelectronic devices [9]. Compared with inorganic compounds [10], metal complexes [11,12], dendrimers [13], and polymers [14], organic luminescent molecules have the advantages of low cost, structural tunability, functional controllability, large-scale production, and rich fluorescent performance, making them promising candidates for mechanoresponsive smart materials [15]. However, traditional organic emitters exhibiting strong fluorescence in solution normally contain planar, rigid, and π-conjugated polycyclic structures, which causes non or weakly emissive aggregated or solid state due to aggregation-caused quenching (ACQ) effects [16,17]. To address this issue, Tang and co-workers accidentally discovered that non-planar-twisted molecular structures exhibited aggregation-induced emissions (AIEs), which could effectively suppress the ACQ phenomenon and lead to the fabrication of efficient solid-state fluorescent materials [18,19]. Meanwhile, the discovery of the AIE phenomenon also provides valuable guidance for mechanochromic organic materials, considering that mechanochromism is the common nature of AIE luminogens [20,21,22].

Cyano-substituted oligo(*p*-phenylene vinylenes) (CN-OPVs) are an important class of conjugated materials with high solid-state efficiency due to the non-planar geometry with reduced π-π stacking interactions [23,24,25,26,27,28,29,30]. Through suitable molecular design, CN-OPVs bearing different functional segments or substituents, such as triphenylamine (TPA) [31,32,33], carbazole [34,35], trifluoromethyl (CF_3_) [36,37], and tetraphenylethene [38], have been developed with excellent mechanochromic properties. Meanwhile, alkyl (alkoxy) length [39,40,41] and position [42], donor–acceptor interactions [43,44,45], supramolecular interactions [46], and crystallinity [47,48] also play important roles in developing smart mechanochromic materials. Their work mechanisms are usually associated with molecular conformation and packing modes, which undergo a phase transition from a crystalline nature to an amorphous phase [49] or between two different crystalline states [50,51]. The variations in solid-state morphologies further affect the intramolecular charge transfer (ICT) [52] and excited-state switching process [53]. During the above process, the energy level of the molecule is affected, and thus, the solid emission color is changed, which can be converted back to the initial state after exposure to either solvent vapor or thermal treatment.

**Scheme 1:**
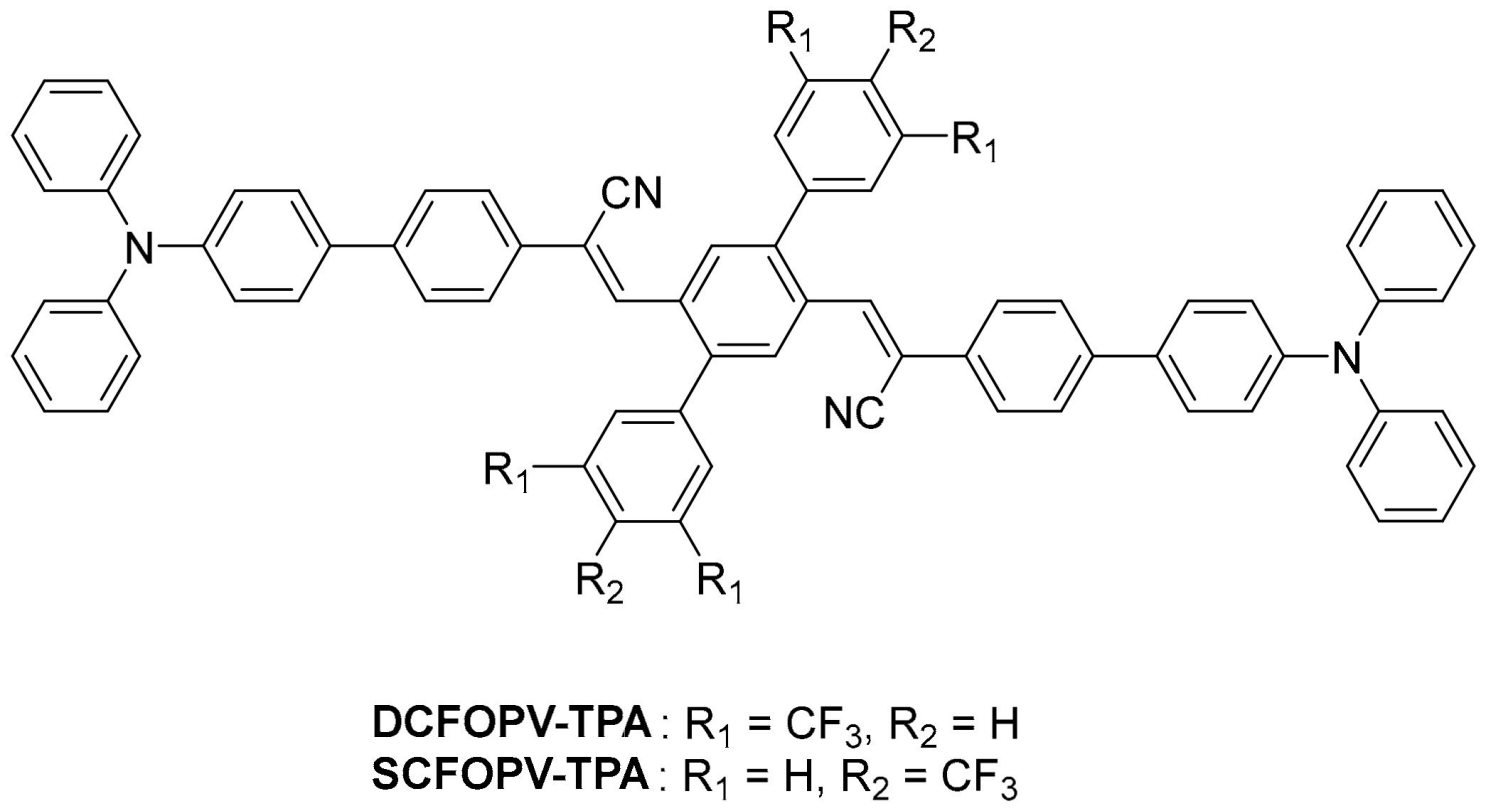
The molecular structures of target compounds.

Organic molecules possessing donor and acceptor moieties are promising candidates for mechanochromic materials due to the possible generation of a twisted intramolecular charge transfer excited state, which is highly sensitive to external mechanical force and can cause shifts in the corresponding photoluminescence spectra [54,55]. Meanwhile, a suitable donor–acceptor functionality in the molecule lowers the optical band gap and results in a red/NIR emission, which is suitable for imaging applications [56]. Our group has reported the self-assembly properties of OPVs via the modulation of the conjugation length, regioregularity, hydrogen bonding, and rigid morphon linker [57,58,59,60]. Recently, we developed solvent-induced multistimuli-responsive properties of CN-OPVs [61]. According to our previous experience and the relevant literature [62,63,64], we herein developed the preparation of two twisted donor–acceptor molecules, DCFOPV-TPA and SCFOPV-TPA, with a different number and position of the CF_3_ group installed (Scheme 1). TPA was utilized as the electron-donating group, which could regulate molecular packing due to the propeller-like structure. Dicyanovinylbenzene was utilized as the electron-withdrawing group, which also allowed for versatile conformational arrangement through twist elasticity. The introduction of CF_3_ groups could further tune the electronic configuration and conformational structure. Both compounds exhibited aggregation-induced enhanced emission (AIEE) behavior in the THF/H_2_O system and bathochromic shift with increasing solvent polarity. The ICT nature of DCFOPV-TPA and SCFOPV-TPA was further confirmed by DFT calculations. Compared with SCFOPV-TPA with no MFC behavior, DCFOPV-TPA displayed an MFC phenomenon with an emission wavelength red-shift of 25 nm, probably due to the larger distortion angle and more pronounced ICT effect of DCFOPV-TPA.

## 2. Results

### 2.1. Chemical Synthesis and Characterization

**Scheme 2:**
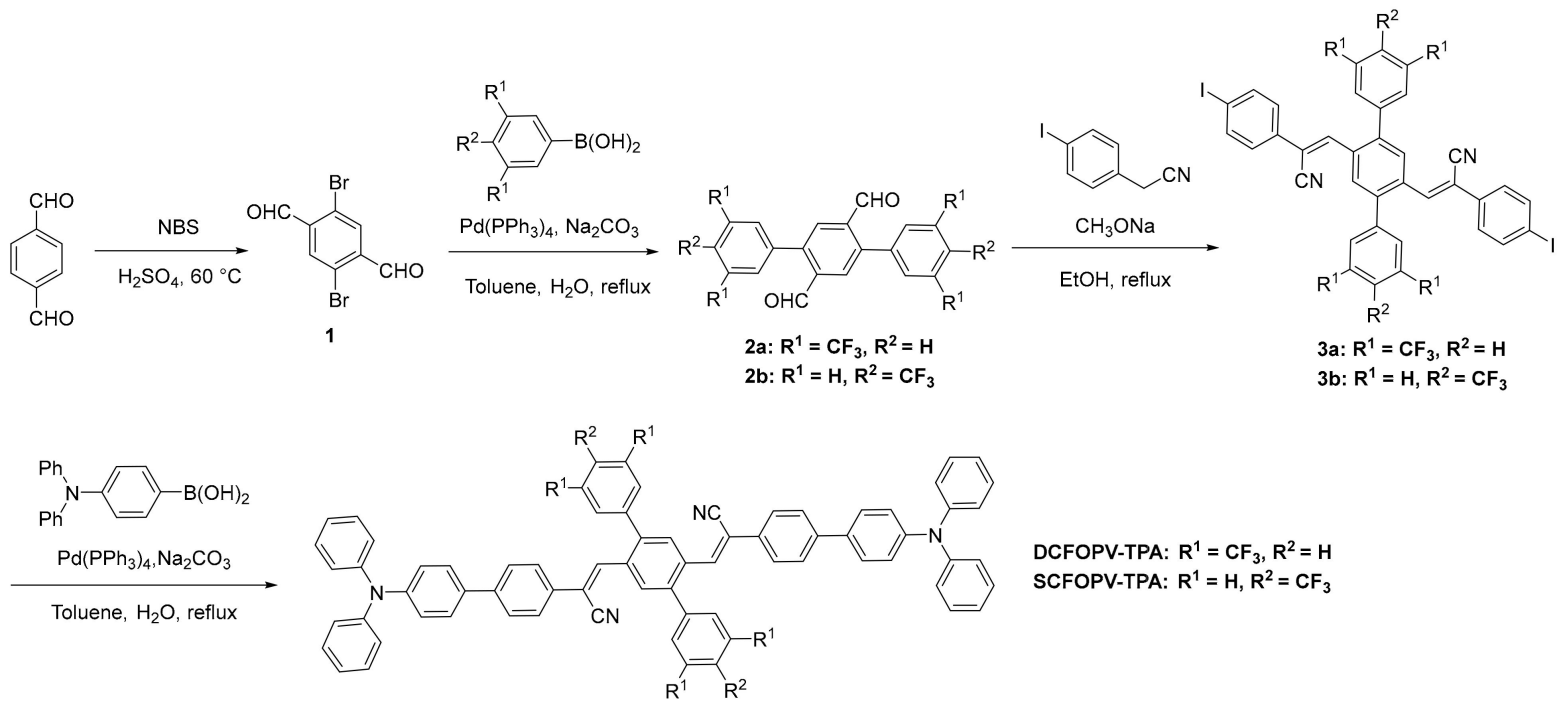
The synthetic route for target molecules.

The synthetic procedure for DCFOPV-TPA and SCFOPV-TPA is described in Scheme 2. Compound **1** was prepared via an electrophilic substitution reaction with NBS according to the previous literature [65]. A subsequent Suzuki–Miyaura cross-coupling reaction between aldehyde **1** and trifluoromethyl-substituted phenyl boronic acids was performed to give the corresponding products **2a** and **2b** 80% and 83% yields, respectively. Next, Knoevenagel condensation between **2** and 2-(4-iodophenyl) acetonitrile using MeONa as the base generated the corresponding products **3a** and **3b** in an up to 85% yield. Finally, the target compounds, DCFOPV-TPA and SCFOPV-TPA, were obtained via a Pd-catalyzed Suzuki–Miyaura cross-coupling reaction in 78% and 79% yields, respectively. All the new compounds were fully characterized by ^1^H NMR, ^13^C NMR, and HRMS analysis (Figures S1–S13).

### 2.2. UV–Vis Absorption and Fluorescent Emission Spectra in Solutions

**Figure 1:**
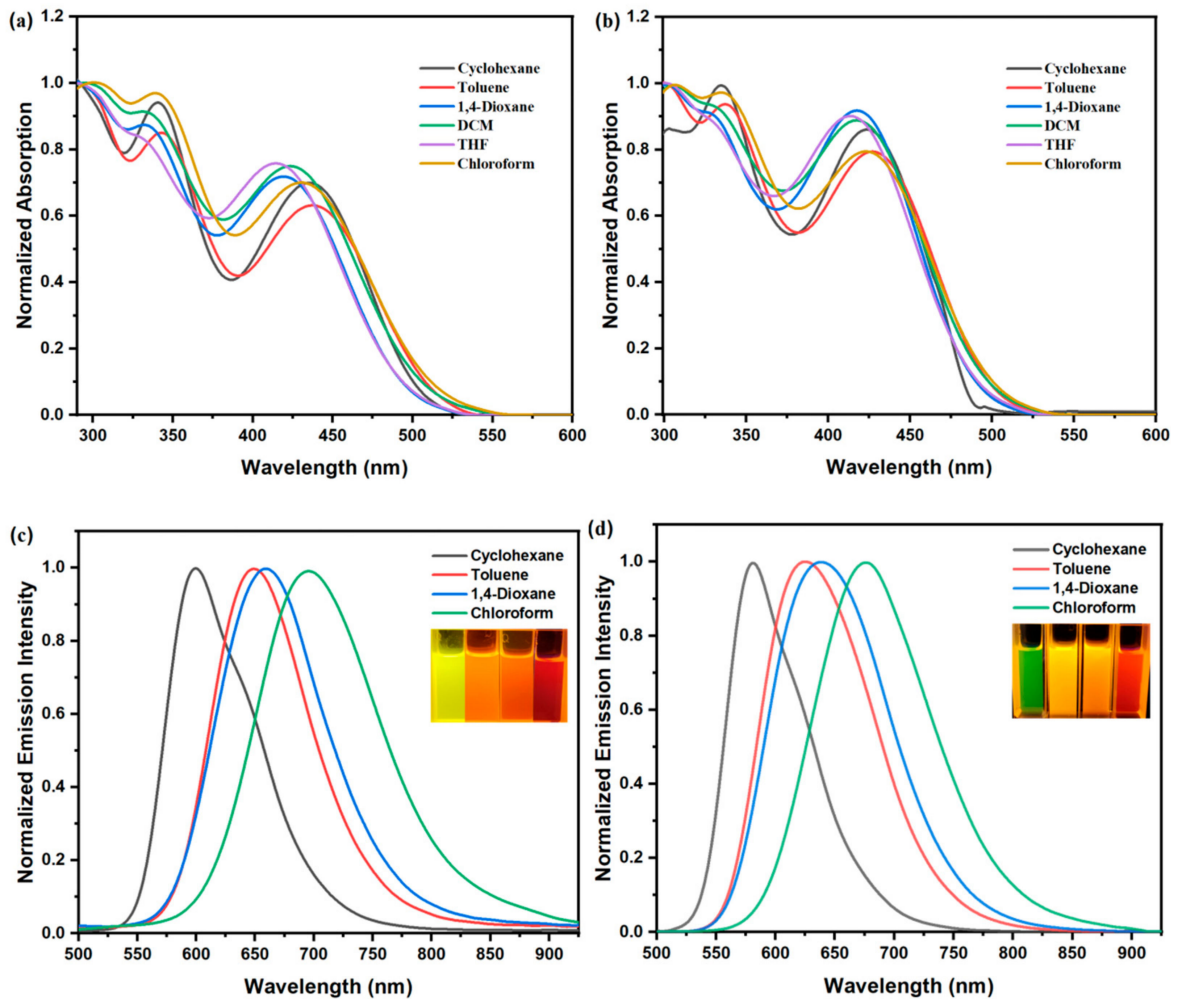
Normalized UV–vis absorption spectra of DCFOPV-TPA (**a**) and SCFOPV-TPA (**b**), and normalized PL spectra of DCFOPV-TPA ((**c**), λ_ex_ = 480 nm) and SCFOPV-TPA ((**d**), λ_ex_ = 480 nm) in different solvents (1.0 × 10^−5^ mol L^−1^). Photos of DCFOPV-TPA and SCFOPV-TPA in different solvents under UV light irradiation (from left to right: cyclohexane, toluene, 1,4-dioxane, chloroform, and λ_ex_ = 365 nm).

The UV-vis absorption and fluorescent emission spectra of DCFOPV-TPA and SCFOPV-TPA in various solvents were initially investigated (Figure 1), and the key photophysical parameters are summarized in Table S1. Due to the inclusion of the electron-donating TPA group and electron-withdrawing cyano moiety, both DCFOPV-TPA and SCFOPV-TPA are donor–acceptor (D-A)-type molecules, which exhibit a typical ICT process. As expected, both compounds exhibited two intense absorption bands in the 280–550 nm region (Figure 1a,b). The absorption peaks located at around 335 nm originated from the π–π* transition, while the absorption bands ranging from 380 nm to 550 nm can be ascribed to the ICT transition from the electron-rich TPA segment to the electron-deficient cyano-substituted vinylbenzene moiety. With the increasing solvent polarity, both DCFOPV-TPA and SCFOPV-TPA exhibit similar absorption profiles with very slightly blue-shifted phenomena, suggesting little difference in the dipole moment in the ground state. For example, in the nonpolar solvents cyclohexane and toluene, the ICT band of DCFOPV-TPA is located at 436 nm and 438 nm, which is blue-shifted to 431 nm, 423 nm, 415 nm, and 419 nm in the polar solvents CHCl_3_, DCM, THF, and 1,4-dioxane. It could be that larger dihedral angles in more polar solvents lead to a lower degree of conjugation, which further causes the absorption peak to be blue-shifted [66,67]. To support this hypothesis, density functional theory (DFT) calculations were performed for DCFOPV-TPA (Table S2). Clearly, the dihedral angles of DCFOPV-TPA in the CHCl_3_, DCM, and THF solvents are larger compared with those in cyclohexane and toluene, which, consequently, leads to the blue-shifted simulated UV-vis absorption peaks.

Figure 1c,d show the fluorescence spectra of the compounds DCFOPV-TPA and SCFOPV-TPA in solvents varying in polarity, which demonstrate prominent solvatochromic behavior, consistent with the strong ICT character of a solvent-relaxed emissive state. By increasing the solvent polarity, a significant bathochromic shift and reduction in the vibration structure in emission spectra were observed, suggesting the enhanced dipole moment of the polarized excited state when stabilized by more polar solvents. For example, in nonpolar cyclohexane, DCFOPV-TPA exhibited yellow light centered at 600 nm, with a vibrational structure appearing as a shoulder in the emission band. With increasing solvent polarity, the emission bands were gradually red-shifted to 649 nm (in toluene), 659 nm (in 1,4-dioxane), and 695 nm (in CHCl_3_). For SCFOPV-TPA, the emission wavelength was located at 581 nm in cyclohexane with a green color, which was red-shifted to 625 nm (in toluene), 638 nm (in 1,4-dioxane), and 676 nm (in CHCl_3_). Compared with SCFOPV-TPA, DCFOPV-TPA exhibited an increased ICT effect, considering the larger maximum ICT absorption and emission bands of DCFOPV-TPA. The fluorescence quantum yielded (Φ_f_) of DCFOPV-TPA and SCFOPV-TPA, which were determined using fluorescein (Φ_f_ = 0.92, 0.1 mol L^−1^) as the standard, and both showed stronger emissions in the nonpolar solvent (0.72 and 0.69 in cyclohexane) than polar solvent (0.20 and 0.08 in CHCl_3_) because of the restricted ICT transitions. The further increasing solvent polarity in THF and DCM led to a completely quenched emission efficiency, indicating a positive solvatokinetic effect.

### 2.3. Theoretical Calculation

**Figure 2:**
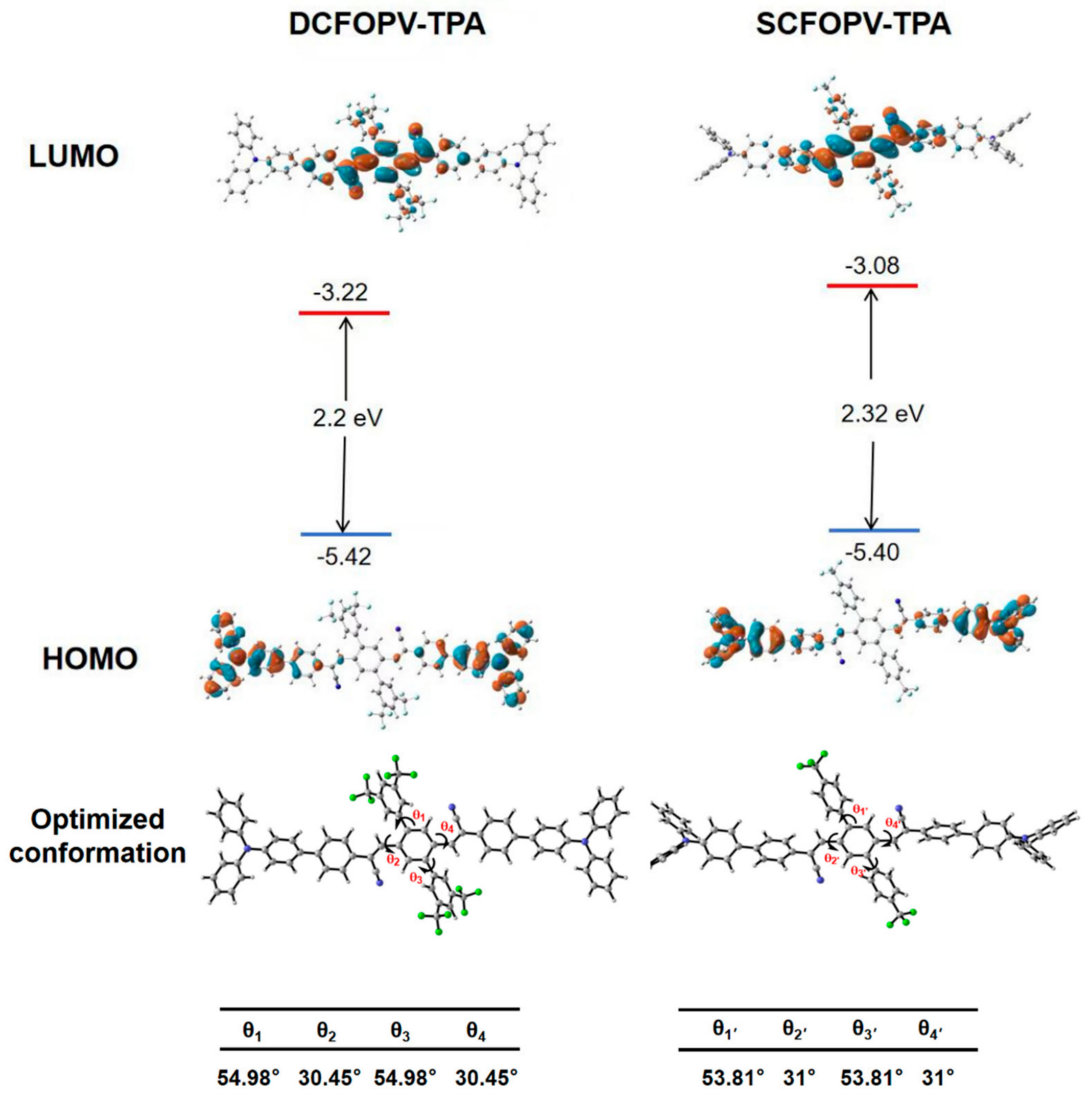
Illustration of spatial electron distribution of HOMOs and LUMOs and optimized molecular geometry of DCFOPV-TPA and SCFOPV-TPA. The DFT calculations were carried out using the Gaussian 16 program at the B3LYP/6-311++G(d, p) level of theory.

To better understand their optical properties, DFT calculations were performed at the B3LYP/6-311++G (d, p) level of theory via the Gaussian 16 program package to clarify the geometric and electronic structures of DCFOPV-TPA and SCFOPV-TPA. As shown in Figure 2, the highest occupied molecular orbitals (HOMOs) of DCFOPV-TPA and SCFOPV-TPA were mainly delocalized on the TPA moiety, while the lowest unoccupied molecular orbitals (LUMOs) were generally distributed over the dicyanovinylbenzene framework, suggesting a typical ICT process. According to their optimized lowest energy state, both DCFOPV-TPA and SCFOPV-TPA adopted distorted molecular conformations, which could suppress close packing arrangements and consequently reduce the effective π-π interaction between neighboring molecules. For DCFOPV-TPA, in detail, the dihedral angles between the central benzene and the vinyl plane were 30.45°. Meanwhile, the dihedral angle between the central core and the CF_3_-substituted benzene arms was 54.89°. Compared with DCFOPV-TPA, the smaller value of 53.81° was observed in the case of SCFOPV-TPA, probably due to the relatively smaller steric hindrance. Such large twist angles in both DCFOPV-TPA and SCFOPV-TPA could be beneficial to AIE characteristics and MFC behavior.

### 2.4. Aggregation-Induced Enhanced Emission (AIEE)

**Figure 3:**
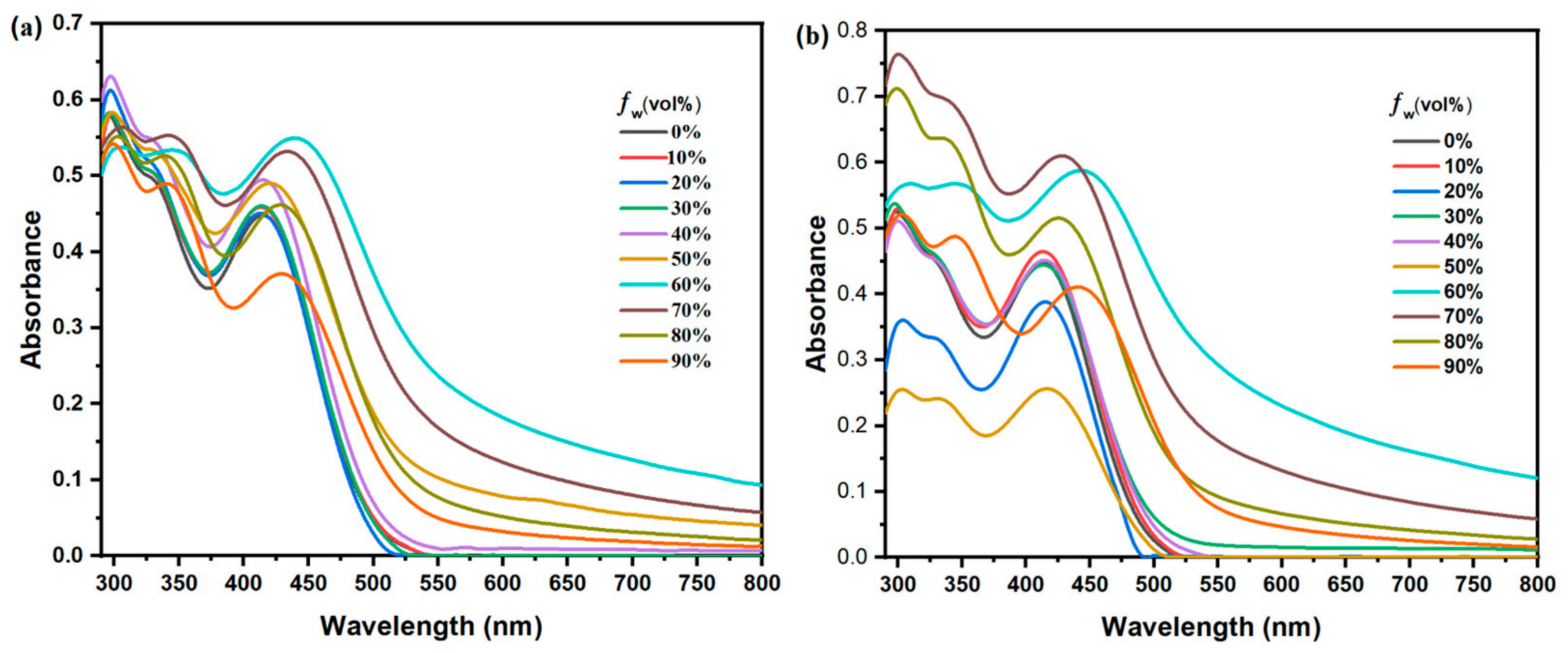
UV–vis absorption spectra of DCFOPV-TPA (**a**) and SCFOPV-TPA (**b**) in THF/water mixtures with different *f_w_*.

CN-OPVs and TPA are common molecular skeletons with excellent aggregation-induced optical properties, which have been regarded as an effective strategy for developing anti-ACQ chromophores. To examine the AIEE behavior of DCFOPV-TPA and SCFOPV-TPA, the UV-vis absorption and fluorescence spectra were investigated in dilute mixtures of THF/water with different volume fractions of water (*f_w_*). As shown in Figure 3, the absorption profiles of DCFOPV-TPA and SCFOPV-TPA showed a significant difference when *f_w_* was above 60%, accompanied by a bathochromic shift from 415 (*f_w_* = 0%) to 429 (*f_w_* = 90%) and from 413 nm (*f_w_* = 0%) to 441 nm (*f_w_* = 90%), respectively. Meanwhile, the appearance of a leveling-off tail in the visible region due to the Mie effect further suggests the formation of nanoaggregation in the solvent mixture (*f_w_* ≥ 60%).

**Figure 4:**
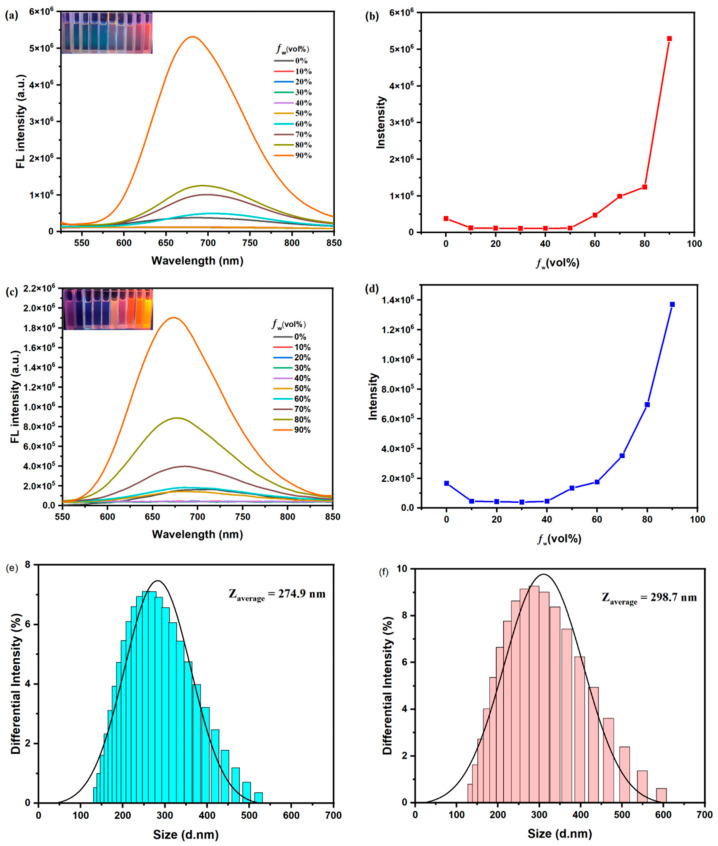
PL spectra of DCFOPV-TPA (**a**), λ_ex_ = 480 nm and SCFOPV-TPA (**c**), λ_ex_ = 480 nm in THF/water mixtures with different *f_w_*. FL intensity versus water fraction of DCFOPV-TPA (**b**) and SCFOPV-TPA (**d**). The insets of (**a**) and (**c**) depict the fluorescence images of DCFOPV-TPA and SCFOPV-TPA with various water fractions (from 0% to 90%, excitation wavelength: 365 nm), respectively. DLS of DCFOPV-TPA (**e**) and SCFOPV-TPA (**f**) in 90% THF/water mixtures. The concentration was 10 μM.

Similarly, both DCFOPV-TPA and SCFOPV-TPA emit weak fluorescence in a pure THF solution excited by light (Figure 4). When *f_w_* is less than 60%, little change in the emission bands is observed, probably caused by the balance between the solvation effect and the molecular aggregation effect. When *f_w_* is above 50–60%, the fluorescence intensity increases significantly due to the formation of nanoaggregates. In the aggregation state, intramolecular rotation and vibration are inhibited. The rotation and vibration of the chemical bonds require energy consumption, and non-radiative dissipation is suppressed, resulting in increased luminous intensity. Further increasing the water fractions (*f_w_*) leads to the gradual enhancement of fluorescence efficiency accompanied by a blue-shifted emission wavelength, ascribed to the encapsulation of DCFOPV-TPA and SCFOPV-TPA in a less polar environment once nanoaggregates are formed. The maximum fluorescence efficiency of DCFOPV-TPA and SCFOPV-TPA was obtained at *f_w_* = 90%, which was about 14-fold and 12-fold, respectively, relative to the original efficiency in a pure THF solution. Finally, according to a dynamic light scattering (DLS) experiment, the average particle sizes (Z_average_) of DCFOPV-TPA and SCFOPV-TPA at the aggregate state (*f_w_* = 90%) were found to be 274.9 and 298.7 nm, respectively, which further confirmed the formation of nanoparticles in solutions.

### 2.5. Mechanofluorochromic (MFC) Properties

**Figure 5:**
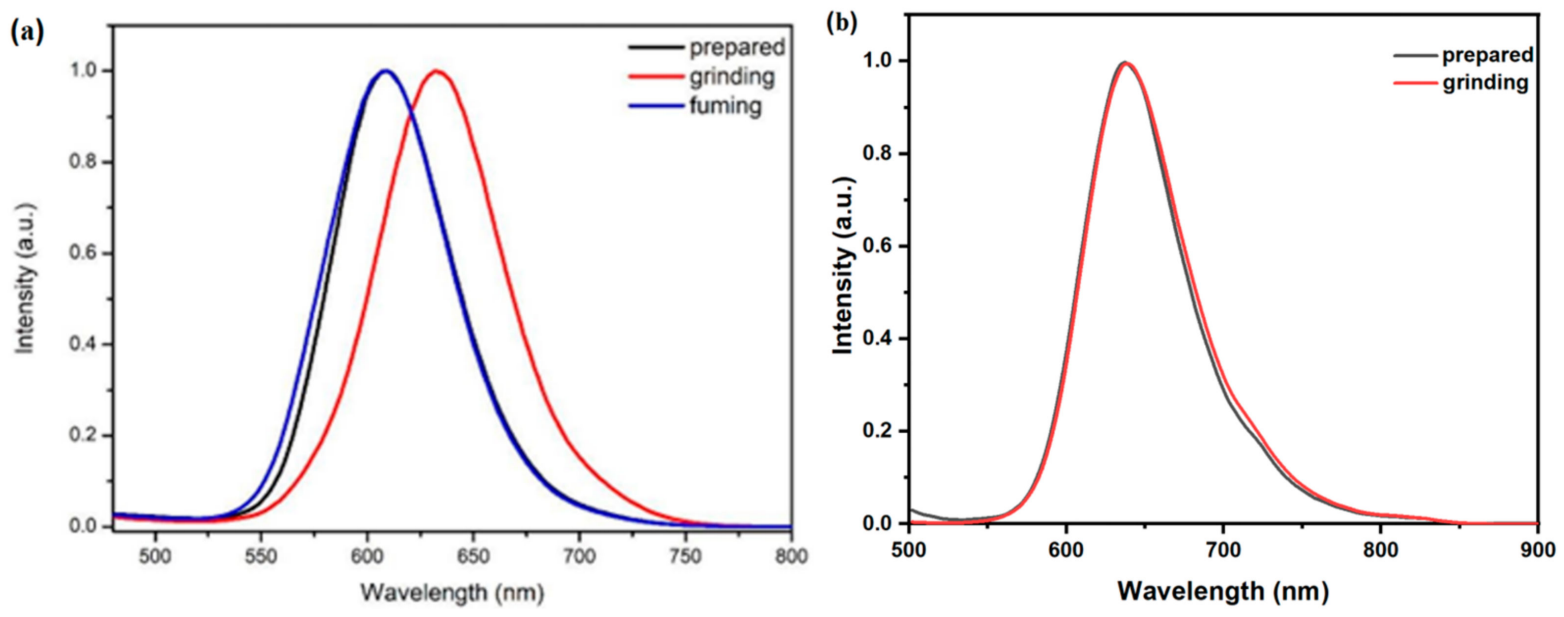
(**a**) Normalized fluorescent spectra of DCFOPV-TPA (**a**) and SCFOPV-TPA (**b**) in the following different solid states: as-prepared, grinding, and fuming (λ_ex_ = 425 nm).

**Figure 6:**
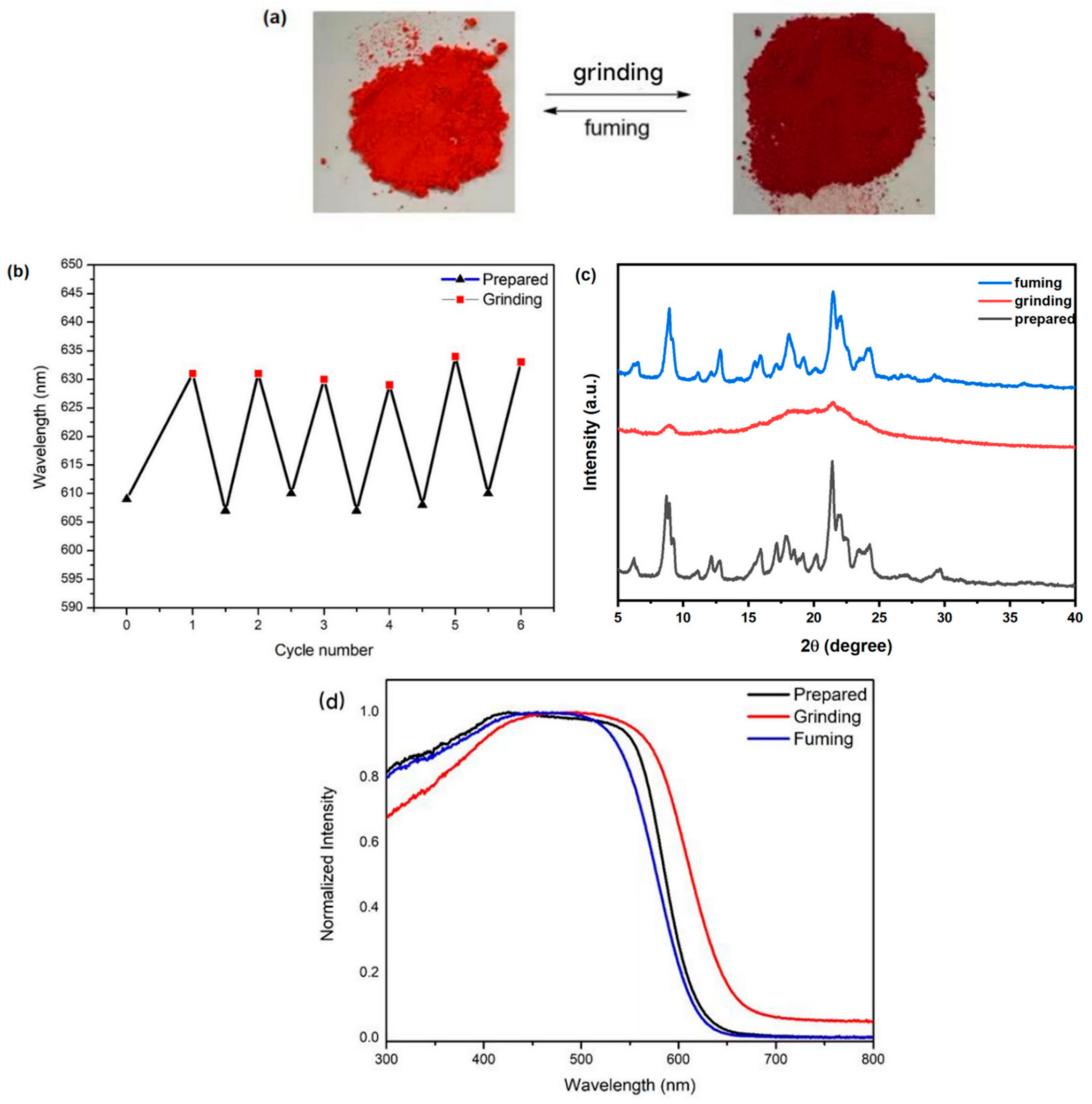
(**a**) Photographs of DCFOPV-TPA color changes under grinding and fuming stimuli. (**b**) Cyclic diagram of stimulus–response performance of DCFOPV-TPA. (**c**) PXRD patterns of DCFOPV-TPA in the following different solid states: as-prepared, grinding, and fuming. (**d**) Normalized UV-vis spectra of DCFOPV-TPA in different states.

Considering the MFC properties of most AIEE chromophores, the fluorescent emission of DCFOPV-TPA and SCFOPV-TPA in the solid was studied (Figure 5 and Figure 6). Upon excitation, both DCFOPV-TPA and SCFOPV-TPA exhibited a bright reddish color, with emission maxima determined to be 607 nm and 639 nm, respectively. Interestingly, only compound DCFOPV-TPA displayed reversible MCF behavior, while SCFOPV-TPA had no MFC properties, probably due to the more steric hindrance nature of DCFOPV-TPA. Specifically, the as-prepared DCFOPV-TPA exhibited an emission peak at 607 nm, which was red-shifted to 632 nm in the ground state with a dark red color. To check the reversibility of MFC behavior, the ground sample of DCFOPV-TPA was fumed with ethyl acetate for 1 min. Luckily, the dark-red-emitting ground powder could be transferred into a reddish color accompanied by a blue-shifted emission wavelength, which corresponded to the as-prepared DCFOPV-TPA solid.

Importantly, the above grinding–fuming process could be repeated several times, suggesting the reversible nature of MFC properties of DCFOPV-TPA under external stimuli. To further clarify the mechanism of the MFC phenomenon, powder X-ray diffraction (PXRD) patterns for DCFOPV-TPA in different solids were examined. As shown in Figure 6c, the as-prepared DCFOPV-TPA solid in PXRD displayed multiple intense and sharp diffraction peaks, suggesting the existence of crystalline forms. On the contrary, very weak and broad diffraction peaks were observed for the ground state of DCFOPV-TPA, suggesting the formation of an amorphous state. It was hypothesized that external force leads to a collapse in the crystalline lattice, leading to red-shifted emissions due to the disordered molecular packing and greater planar conformation. Compared with solid absorption peaks of as-prepared (425 nm) DCFOPV-TPA, the grinding state was red-shifted to 447 nm, further proving a more effective conjugation length after grinding.

## 3. Materials and Methods

### 3.1. General Methods

All chemicals were commercially available and utilized without further purification unless otherwise specified. Column chromatography was performed using 200–300 mesh silica gel. ^1^H NMR, ^13^C NMR, and ^19^F NMR spectra were recorded on a Bruker DPX 600 instrument (Brooke Technologies Co., Ltd., Zurich, Switzerland) using TMS as the internal standard. HRMS was determined on a waters UPLC G2-XS Qtof system ESI spectrometer (Waters World Science & Technology Co., Ltd., Wilmslow, UK). The UV-vis absorption and fluorescent emission spectra were assessed by a dual-beam UV-vis spectrophotometer (TU-1901) (Agilent, Inc., Santa Clara, CA, USA) and Hitachi F-7000 spectrofluorimeter (Hitachi High-tech (Shanghai) International Trade Co., Ltd., Shanghai, China), respectively. Powder X-ray diffraction (PXRD) was performed on an X’Pert PRO Powder X-ray diffraction instrument (Holland PANalytical, Almelo, The Netherlands). The DFT calculations were carried out using the Gaussian 16 program at the B3LYP/6-311++G (d, p) level of theory, and the solvent effects of THF were simulated by the SMD mode.

### 3.2. General Procedure for Preparation of 2,5-Dibromoterephthalaldehyde ***1***

A 100 mL two-necked round-bottom flask had terephthalaldehyde (3.0 g, 22.39 mmol) added to it in concentrated H_2_SO_4_ (30 mL), which was heated at 60 °C, followed by the addition of NBS (7.97 g, 44.78 mmol) after 15 min. After stirring at 60 °C for 3 h, the reaction mixture was poured into ice water (30 mL), and the precipitation was filtered. Next, the precipitation was redissolved in CH_2_Cl_2_ (30 mL), which was washed with NaHCO_3_ three times. After the removal of the organic solvent, the crude product was purified via recrystallization in CHCl_3_ to yield compound **1** as the yellow solid (2.61 g, 40% yield). Compound **1** was a known compound, and ^1^H NMR data were the same as in the previous report [65]. ^1^H NMR (600 MHz, CDCl_3_): *δ* ppm 10.35 (s, 2H), 8.15 (s, 2H).

### 3.3. General Procedure for Preparation of Compound ***2***

To a 100 mL Schlenk flask, compound **1** (0.5 g, 1.71 mmol), (3,5-bis(trifluoromethyl)phenyl)boronic acid (1.25 g, 4.79 mmol) or (4-(trifluoromethyl)phenyl)boronic acid (0.91 g, 4.79 mmol), Pd(PPh_3_)_4_ (18 mg, 0.015 mmol) and Na_2_CO_3_ (997 mg, 9.41 mmol) were added in a mixed solvent of toluene and water (37.5 mL, *v*/*v* = 3:1) under Ar. After refluxing for 24 h, the reaction mixture was cooled down to room temperature and the organic layer was collected. The aqueous layer was washed with CH_2_Cl_2_ three times. The organic layer was combined and concentrated. After the removal of the organic solvent, the organic residue was purified via column chromatography using petroleum ether/CH_2_Cl_2_ (*v*/*v* = 2/1) as an eluent to afford the corresponding products **2a** and **2b**. Compound **2** was a known compound, and ^1^H NMR data were the same as in the previous report [68].

**2a**: white solid (763 mg, 80% yield). ^1^H NMR (600 MHz, CDCl_3_): *δ* ppm 10.06 (s, 2H), 8.15 (s, 2H), 8.06 (s, 2H), 7.91 (s, 4H).

**2b**: white solid (599 mg, 83% yield). ^1^H NMR (600 MHz, CDCl_3_): *δ* ppm 10.07 (s, 2H), 8.12 (s, 2H), 7.81 (d, *J* = 8.0 Hz, 4H), 7.59 (d, *J* = 8.0 Hz, 4H).

### 3.4. General Procedure for Preparation of Compounds ***3***

To a 100 mL Schlenk flask, compound **2a** (1.0 g, 1.79 mmol) or **2b** (756 mg, 1.79 mmol) and 2-(4-iodophenyl)acetonitrile (1.25 g, 4.79 mmol) were added in dry EtOH (40 mL) under Ar, followed by the addition of MeONa (193 mg, 3.58 mmol). After refluxing for 8 h, the reaction mixture was cooled down to room temperature, and the precipitation was filtered. The obtained precipitation was washed with water, EtOH, and warm EtOAc in sequence to afford the corresponding products **3a** and **3b**.

**3a**: yellowish green solid (1.53 g, 85% yield). m.p.: 280 °C. ^1^H NMR (600 MHz, CDCl_3_): *δ* ppm 8.30 (s, 2H), 8.02 (s, 2H), 7.97 (s, 4H), 7.78 (d, *J* = 8.6 Hz, 4H), 7.43 (s, 2H), 7.29 (d, *J* = 8.6 Hz, 4H). HR-MS (ESI, *m*/*z*): [M + K]^+^ calcd for [C_40_H_18_I_2_F_12_N_2_K]^+^, 1046.8999. found, 1046.9003.

**3b**: yellowish green solid (1.12 g, 72% yield). m.p.: 292 °C. ^1^H NMR (600 MHz, CDCl_3_): *δ* ppm 8.25 (s, 2H), 7.82–7.73 (m, 8H), 7.62 (d, *J* = 8.0 Hz, 4H), 7.48 (s, 2H), 7.30 (d, *J* = 8.5 Hz, 4H). HR-MS (ESI, *m*/*z*): [M + Na]^+^ calcd for [C_38_H_20_F_6_I_2_N_2_Na]^+^, 894.9512. found, 894.9512.

### 3.5. General Procedure for Preparation of the Compounds DCFOPV-TPA and SCFOPV-TPA

To a 100 mL Schlenk flask, compound **3a** (800 mg, 0.79 mmol) or **3b** (1.56 g, 1.79 mmol), (4-(diphenylamino)phenyl)boronic acid (639 mg, 2.21 mmol), Pd(PPh_3_)_4_ (27.39 mg, 0.02 mmol) and Na_2_CO_3_ (460 mg, 4.35 mmol) were added in a mixed solvent of toluene and water (64 mL, *v*/*v* = 3:1) under Ar. After refluxing for 24 h, the reaction mixture was cooled down to room temperature, and the organic layer was collected. The aqueous layer was washed with CH_2_Cl_2_ three times. The organic layer was combined and concentrated. After removal of the organic solvent, the organic residue was purified via column chromatography using petroleum ether/CH_2_Cl_2_ (*v*/*v* = 2/1) as an eluent to afford the corresponding products DCFOPV-TPA and SCFOPV-TPA.

DCFOPV-TPA: red solid (765 mg, 78% yield). m.p.: 305 °C. ^1^H NMR (600 MHz, CDCl_3_): *δ* ppm 8.36 (s, 2H), 8.05 (d, *J* = 3.3 Hz, 6H), 7.68–7.63 (m, 8H), 7.53–7.50 (m, 4H), 7.49 (s, 2H), 7.31 (dd, *J* = 8.4, 7.5 Hz, 8H), 7.17 (dd, *J* = 8.5, 1.2 Hz, 12H), 7.08 (t, *J* = 7.4 Hz, 4H). ^13^C NMR (151 MHz, CDCl_3_) *δ* ppm 147.4, 142.5, 140.5, 139.3, 137.2, 134.0, 133.0, 132.7, 132.7, 132.5, 132.3, 131.1, 131.0, 129.4, 127.7, 127.2, 126.6, 125.8, 124.7, 124.0, 123.4, 123.3, 122.4, 122.1, 120.3, 117.5, 116.7, 100.0, 77.2, 77.0, 76.8. HRMS (ESI, *m*/*z*): [M + Na]^+^ calcd for [C_76_H_46_F_12_N_4_Na]^+^, 1265.3423. found, 1265.3424.

SCFOPV-TPA: yellowish green solid (1.56 g, 79% yield). m.p.: >320 °C. ^1^H NMR (600 MHz, CDCl_3_): *δ* ppm 8.29 (s, 2H), 7.79 (d, *J* = 8.1 Hz, 4H), 7.70–7.62 (m, 12H), 7.52 (s, 2H), 7.49 (d, *J* = 8.6 Hz, 4H), 7.28 (t, *J* = 7.9 Hz, 8H), 7.14 (dd, *J* = 8.2, 3.1 Hz, 12H), 7.06 (t, *J* = 7.4 Hz, 4H). ^13^C NMR (151 MHz, CDCl_3_) *δ* ppm 147.5, 142.2, 140.7, 138.9, 133.8, 133.1, 131.7, 130.9, 130.4, 129.4, 127.7, 127.2, 126.5, 125.8, 124.8, 123.4, 123.3, 117.8, 115.0. 77.2, 77.0, 76.8. HRMS (ESI, *m*/*z*): [M + Na]^+^ calcd for [C_74_H_48_F_6_N_4_Na]^+^, 1107.3856. found, 1107.3865.

## 4. Conclusions

In conclusion, two novel CN-substituted OPVs, DCFOPV-TPA and SCFOPV-TPA, equipped with TPA and CF_3_ segments, were designed and synthesized to investigate their photophysical properties. Both DCFOPV-TPA and SCFOPV-TPA exhibited a typical AIEE effect. Also, remarkable solvatochromic behavior was observed due to the highly distorted conformation and ICT transition within the D-A-type molecules. Moreover, DCFOPV-TPA demonstrated reversible MCF phenomena under external force due to phase transition from the crystalline to the amorphous state. The AIEE features and MCF performance of DCFOPV-TPA are beneficial for the development of smart materials with potential applications in mechanical sensors, data encryption, and security protection.

## Data Availability

The data presented in this study are available in the Supplementary Materials.

## Supplementary Materials

The following supporting information can be downloaded at: https://www.mdpi.com/article/10.3390/molecules29184447/s1, Figures S1–S13: ^1^H NMR, ^13^C NMR, and HRMS analysis; Table S1: Photophysical properties of compounds DCFOPV-TPA and SCFOPV-TPA; Table S2: Optimized molecular geometry of DCFOPV-TPA in different solvents. Ref. [69] is cited in the Supplementary Materials.
