# Supplementary material for: Cyano-Substituted Oligo(p-phenylene vinylene) Derivatives with Aggregation-Induced Enhanced Emissions and Mechanofluorochromic Luminescence
Source: Molecules. 2024 Sep 19;29(18):4447. doi: 10.3390/molecules29184447 (PMC11434572; doi:10.3390/molecules29184447)
Supplement: Supplementary file 1 [file molecules-29-04447-s001.zip › molecules-3141244-supplementary.pdf]

Electronic Supplementary Information (ESI)

Cyano-Substituted Oligo(*p*-phenylene vinylene) Derivatives with Aggregation-Induced Enhanced Emissions and Mechanofluorochromic Luminescence

Xinju Zhu *,†, Yaru Pan †, Xinran Zhao †, Yu Yuan, Zewen Zhai, Xiaoni Yu, Wenjing Zhang, Yuanyuan Chang *, Bing Song, Linlin Shi and Xinqi Hao *

College of Chemistry, Zhengzhou University, No. 100 of Science Road, Zhengzhou 450001, China; pyr12042023@163.com (Y.P.); zxr6121@163.com (X.Z.); 19561343255@163.com (Y.Y.); zzwen0403@163.com (Z.Z.); yuxn0802@163.com (X.Y.); zhangwj@zzu.edu.cn (W.Z.); bingsong@zzu.edu.cn (B.S.); linlinshi@zzu.edu.cn (L.S.)

* Correspondence: zhuxinju@zzu.edu.cn (X.Z.); changyy2018@zzu.edu.cn (Y.C.); xqhao@zzu.edu.cn (X.H.)

† These authors contributed equally to this work.

Table of Contents

1. Photophysical Data..3

2. DFT Calculations..4

3. NMR Spectra..31

4. HRMS Spectra 36

Photophysical Data

Table S1. Photophysical properties of compounds **DCFOPV-TPA** and **SCFOPV-TPA**

Sample	Solvent	Δf	λ_{abs}/nm(ε/ M^{-1}•cm^{-1})	λ_{em}/nm	$\Delta \upsilon_{st}{}^{a}$/$cm^{-1}$	$\Phi_f{}^{b}$[%]
**DCFOPV-TPA**	Cyclohexane	0.006	290(43780)，341(41310)，436(30630)	600	6269	72
	Toluene	0.01321	294(52950)，343(45140)，438(33460)	649	7422	56
	1,4-Dioxane	0.02465	292(59510)，333(52210)，419(42770)	659	8691	48
	Chloroform	0.14829	295(57930)，337(56600)，431(40730)	695	8813	20
	DCM	0.21717	293(62810)，332(57570)，423(47100)	-	-	-
	THF	0.20964	293(64660)，325(54680)，415(49050)	-	-	-
**SCFOPV-TPA**	Cyclohexane	0.006	303(2550)，334(3000)，426(2590)	581	6262	69
	Toluene	0.01321	299(43130)，337(40690)，427(34260)	625	7419	54
	1,4-Dioxane	0.02465	302(50900)，325(46570)，418(46750)	638	8249	50
	Chloroform	0.14829	308(46290)，335(45230)，423(36800)	676	8847	8
	DCM	0.21717	306(48490)，326(45460)，418(43120)	-	-	-
	THF	0.20964	304(47150)，324(42580)，413(42530)	-	-	-

^{a}Calculated via υ_{abs}-υ_{em}. ^{b}Quantum yield values in the above mentioned solvents were measured with respect to quinine sulphate in 0.1 M H_2SO_4 ($\varphi_{quinine\ sulphate}$ = 0.55).

DFT Calculations

Table S2. Optimized molecular geometry of **DCFOPV-TPA** in different solvents[a]

ε	solvents	θ_1	θ_2	θ_3	θ_4	λ (nm)
2.02	cyclohexane	-54.41	-33.24	-54.41	-33.24	440.7
2.21	1,4-dioxane	-54.38	-33.57	-54.38	-33.57	440.1
2.37	toluene	-54.35	-33.92	-54.35	-33.92	439.9
4.71	chloroform	-54.14	-36.11	-54.14	-36.11	436.0
7.43	THF	-53.57	-36.99	-53.57	-36.99	433.2
8.93	DCM	-53.93	-36.95	-53.93	-36.95	433.0

[a]The structures were optimized using the Gaussian 16 program at the B3LYP/6-311++G(d,p)/IEFPCM level of theory.

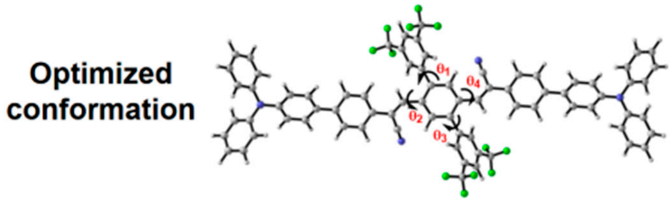

Complete Reference for Gaussian 16

Frisch MJ, Trucks GW, Schlegel HB, Scuseria GE, Robb MA, Cheeseman JR, Scalmani GB, V., Petersson GA, Nakatsuji HL, X., Caricato M, Marenich AV, Bloino J, Janesko BG, Gomperts R, Mennucci B, Hratchian HP, Ortiz JV, Izmaylov AF, Sonnenberg JL, Williams-Young D, Ding F, Lipparini F, Egidi F, Goings J, Peng B, Petrone A, Henderson T, Ranasinghe D, Zakrzewski VG, Gao J, Rega N, Zheng G, Liang W, Hada M, Ehara M, Toyota K, Fukuda R, Hasegawa J, Ishida M, Nakajima T, Honda Y, Kitao O, Nakai H, Vreven T, Throssell K, Montgomery JA, Jr., Peralta JE, Ogliaro F, Bearpark MJ, Heyd JJ, Brothers EN, Kudin KN, Staroverov VN, Keith TA, Kobayashi R, Normand J, Raghavachari K, Rendell AP, Burant JC, Iyengar SS, Tomasi J, Cossi M, Millam JM, Klene M, Adamo C, Cammi R, Ochterski JW, Martin RL, Morokuma K, Farkas O, Foresman JB, Fox DJ. *Gaussian 16*, Gaussian, Inc., Wallingford CT, 2016.

Cartesian coordinates of all structures involved.

DCFOPV-TPA

0 1

C	0.63184400	1.22419400	–0.30456700
C	–0.75999400	1.18592800	–0.31083600
C	–1.42618100	–0.06693600	–0.31425700
C	–0.63185200	–1.22415000	–0.30465500
C	0.75998600	–1.18588300	–0.31092100
C	1.42617200	0.06698100	–0.31425300
H	1.11125900	2.19281900	–0.29000100
H	–1.11126700	–2.19277700	–0.29015800
C	2.88644000	0.12118700	–0.23345900
C	3.75493400	1.07041500	–0.67121200
H	3.33989500	–0.73382400	0.25395400
C	–2.88644900	–0.12115200	–0.23346200
C	–3.75494000	–1.07035500	–0.67127100
H	–3.33990500	0.73382400	0.25401000
C	3.29460400	2.23819700	–1.36436300
N	2.96150000	3.19152100	–1.92763500
C	–3.29460700	–2.23809400	–1.36449300
N	–2.96149800	–3.19138600	–1.92781600
C	5.22535800	0.97278500	–0.49571300
C	6.02205600	2.12797400	–0.50388600
C	5.86891200	–0.26309900	–0.31974100
C	7.39504900	2.05312500	–0.31178200
H	5.56068100	3.09864900	–0.64371700
C	7.24123400	–0.33434300	–0.13581200
H	5.30518000	–1.18676100	–0.36795500
C	8.04068100	0.82202800	–0.12020100
H	7.97131800	2.97043100	–0.28698100
H	7.70717600	–1.30837000	–0.04465200
C	–5.22536400	–0.97274400	–0.49575800
C	–6.02205500	–2.12793700	–0.50399300
C	–5.86892500	0.26312600	–0.31971400
C	–7.39504800	–2.05310700	–0.31187700
H	–5.56067400	–3.09860100	–0.64388100
C	–7.24124600	0.33435200	–0.13577500
H	–5.30519900	1.18679400	–0.36788000
C	–8.04068600	–0.82202500	–0.12022400
H	–7.97131100	–2.97041800	–0.28712600
H	–7.70719400	1.30837100	–0.04455800
C	9.50400900	0.74044400	0.07529400
C	10.38021900	1.61895900	–0.58169800
C	10.07500300	–0.21959800	0.92579900
C	11.75473100	1.54059500	–0.40774300
H	9.98455300	2.35140000	–1.27617700

C	11.44701300	–0.29581500	1.11968400
H	9.43288000	–0.89494700	1.47990000
C	12.31445700	0.58212200	0.45135000
H	12.40426300	2.21785600	–0.94825700
H	11.85356500	–1.03330100	1.80054100
C	–9.50401400	–0.74046100	0.07528300
C	–10.38022100	–1.61894500	–0.58175300
C	–10.07500900	0.21953000	0.92584400
C	–11.75473300	–1.54060000	–0.40778700
H	–9.98455400	–2.35134600	–1.27627400
C	–11.44701900	0.29572800	1.11974000
H	–9.43288800	0.89485300	1.47997900
C	–12.31446100	–0.58217800	0.45136200
H	–12.40426400	–2.21783600	–0.94833400
H	–11.85357200	1.03317400	1.80063900
C	–1.49529400	2.48221100	–0.32881800
C	–2.41115300	2.78026700	–1.34454400
C	–1.24496200	3.44894700	0.65124500
C	–3.05978700	4.01295400	–1.37231300
H	–2.59667600	2.05904100	–2.13060700
C	–1.89780200	4.67926100	0.61663900
H	–0.53896300	3.23825600	1.44489100
C	–2.81193300	4.97058600	–0.39205500
H	–3.30921200	5.93064100	–0.42302300
C	1.49528800	–2.48216300	–0.32899400
C	2.41116200	–2.78013800	–1.34473100
C	1.24495200	–3.44897300	0.65099600
C	3.05980200	–4.01282000	–1.37258500
H	2.59669100	–2.05885100	–2.13073600
C	1.89779900	–4.67928100	0.61630400
H	0.53894400	–3.23834500	1.44465000
C	2.81194100	–4.97052600	–0.39240300
H	3.30922600	–5.93057600	–0.42343600
C	1.65646900	–5.67973400	1.71826700
C	–1.65647700	5.67962900	1.71868000
C	4.07964500	–4.28958500	–2.44788800
C	–4.07961600	4.28980600	–2.44760700
F	1.86372800	–6.94795500	1.30839100
F	–1.86375600	6.94788000	1.30890700
F	2.48785200	–5.46664900	2.76952600
F	–2.48784800	5.46644900	2.76992900
F	0.39724900	–5.61006200	2.19979100
F	–0.39725200	5.60993400	2.20018800
F	4.21064100	–5.60742900	–2.70102000

F	–4.21059800	5.60767000	–2.70064500
F	5.30927400	–3.83694700	–2.08506500
F	–5.30925200	3.83715300	–2.08482900
F	3.77126900	–3.68065600	–3.61095500
F	–3.77123300	3.68095900	–3.61071500
N	13.71355100	0.50245800	0.63774700
N	–13.71355400	–0.50253400	0.63777100
C	14.50294100	1.68700300	0.66917600
C	15.71742800	1.74774800	–0.02678400
C	14.08141900	2.80498000	1.40139600
C	16.49381600	2.90225000	0.01561800
H	16.04896000	0.88788700	–0.59626900
C	14.85592400	3.96110400	1.42544400
H	13.14734100	2.76301700	1.94855600
C	16.06738300	4.01681300	0.73688200
H	17.43084900	2.93345900	–0.52922000
H	14.51639500	4.81721300	1.99790800
H	16.67126000	4.91628900	0.76295200
C	–14.50293700	–1.68708500	0.66913600
C	–15.71742600	–1.74779700	–0.02682200
C	–14.08140400	–2.80510200	1.40128900
C	–16.49380700	–2.90230700	0.01551800
H	–16.04896700	–0.88790600	–0.59625500
C	–14.85590100	–3.96123200	1.42527300
H	–13.14732400	–2.76316400	1.94844600
C	–16.06736300	–4.01690900	0.73671500
H	–17.43084300	–2.93349000	–0.52931700
H	–14.51636500	–4.81737200	1.99768600
H	–16.67123500	–4.91639000	0.76273500
C	14.34664500	–0.76338700	0.79365800
C	14.00756800	–1.83734400	–0.04023100
C	15.32416500	–0.94977300	1.77994700
C	14.62769000	–3.07305700	0.11956700
H	13.25831900	–1.69894900	–0.81037400
C	15.95107600	–2.18433500	1.92247200
H	15.59053300	–0.12439700	2.42910600
C	15.60480100	–3.25421200	1.09798800
H	14.35514600	–3.89367100	–0.53480900
H	16.70518200	–2.31246000	2.69109100
H	16.09056000	–4.21581100	1.21535900
C	–14.34665500	0.76329900	0.79375800
C	–14.00759000	1.83730500	–0.04007200
C	–15.32416900	0.94962300	1.78006400
C	–14.62771900	3.07300600	0.11980100

H	–13.25834500	1.69895900	–0.81022700
C	–15.95108700	2.18417200	1.92266500
H	–15.59052800	0.12420800	2.42917800
C	–15.60482400	3.25409800	1.09823900
H	–14.35518400	3.89365800	–0.53453000
H	–16.70518900	2.31224800	2.69129500
H	–16.09058900	4.21568700	1.21566900

SCFOPV-TPA

0 1

C	0.63188100	–1.22468300	–0.41389500
C	–0.76027100	–1.19143000	–0.42170800
C	–1.42202800	0.06467800	–0.42219100
C	–0.63187100	1.22481000	–0.41387700
C	0.76028000	1.19155600	–0.42168500
C	1.42203500	–0.06455000	–0.42219000
H	1.11568500	–2.19168500	–0.39821700
H	–1.11567200	2.19181300	–0.39817800
C	2.88018600	–0.12096500	–0.33816500
C	3.74536500	–1.07013800	–0.78476700
H	3.33776700	0.73917700	0.13628600
C	–2.88017700	0.12108600	–0.33814900
C	–3.74538100	1.07025400	–0.78471500
H	–3.33773900	–0.73907200	0.13629000
C	3.29446000	–2.17601900	–1.57746300
N	2.97764500	–3.07548600	–2.23167600
C	–3.29450000	2.17615400	–1.57740000
N	–2.97769900	3.07563300	–2.23160300
C	5.20781800	–1.00100500	–0.54750700
C	6.10879900	–1.63415400	–1.41733000
C	5.73971500	–0.29856900	0.54608900
C	7.48001600	–1.53943400	–1.22124600
H	5.73316400	–2.19138500	–2.26775000
C	7.11009500	–0.20911600	0.74053800
H	5.07779600	0.15309600	1.27555900
C	8.01700800	–0.82202500	–0.14107200
H	8.14539700	–2.01066300	–1.93505400
H	7.48351000	0.31202000	1.61430600
C	–5.20782800	1.00109200	–0.54744300
C	–6.10882100	1.63432900	–1.41719000
C	–5.73971800	0.29853900	0.54608300
C	–7.48003700	1.53958300	–1.22110700
H	–5.73319800	2.19165300	–2.26755500
C	–7.11009500	0.20906000	0.74053100

H	–5.07779800	–0.15320300	1.27550500
C	–8.01702200	0.82205400	–0.14100900
H	–8.14542000	2.01088600	–1.93486400
H	–7.48349700	–0.31216900	1.61424900
C	9.47765300	–0.72452700	0.07011000
C	10.33457400	–1.79360700	–0.23604300
C	10.06469500	0.44269500	0.58390000
C	11.70498200	–1.70811200	–0.03384900
H	9.91924800	–2.72622900	–0.60087000
C	11.43569100	0.54424800	0.77476700
H	9.44379000	1.30489300	0.79978100
C	12.28216300	–0.53333400	0.47225900
H	12.33580700	–2.55912600	–0.25900800
H	11.85931400	1.46730500	1.15075300
C	–9.47766200	0.72451100	0.07016800
C	–10.33461500	1.79358400	–0.23592000
C	–10.06467800	–0.44275700	0.58388700
C	–11.70502100	1.70804000	–0.03373500
H	–9.91931800	2.72624100	–0.60069100
C	–11.43566900	–0.54435900	0.77474700
H	–9.44375400	–1.30495500	0.79971200
C	–12.28217600	0.53321800	0.47230300
H	–12.33586400	2.55905300	–0.25884800
H	–11.85926500	–1.46745400	1.15067100
C	–1.49948200	–2.48422600	–0.43864200
C	–1.23646800	–3.46297900	0.52975200
C	–2.43999400	–2.76991600	–1.43849400
C	–1.89992000	–4.68467600	0.51014300
H	–0.51378300	–3.26117900	1.31181100
C	–3.10374700	–3.99130900	–1.46603700
H	–2.63732100	–2.03960900	–2.21397500
C	–2.83582100	–4.95008800	–0.48916900
C	1.49950300	2.48434600	–0.43858600
C	1.23649000	3.46308100	0.52982400
C	2.44003900	2.77003700	–1.43841600
C	1.89996100	4.68477000	0.51024900
H	0.51378900	3.26127700	1.31186700
C	3.10381000	3.99141900	–1.46592400
H	2.63736900	2.03973800	–2.21390300
C	2.83588100	4.95018600	–0.48904200
N	13.67921100	–0.43814200	0.67159300
N	–13.67921800	0.43796900	0.67160400
C	14.57852800	–1.04227400	–0.25193800
C	15.68670200	–1.76598700	0.20796000

C	14.37318300	–0.91668900	–1.63248500
C	16.57155000	–2.34534800	–0.69689900
H	15.85109400	–1.86883100	1.27378700
C	15.25383300	–1.51278600	–2.53035400
H	13.52308000	–0.35214500	–1.99610300
C	16.35954200	–2.22715300	–2.07001300
H	17.42391600	–2.90283400	–0.32465500
H	15.08160000	–1.40494300	–3.59554100
H	17.04701800	–2.68453800	–2.77185200
C	–14.57851200	1.04226700	–0.25186400
C	–15.68651200	1.76617900	0.20812200
C	–14.37328800	0.91662900	–1.63242000
C	–16.57132800	2.34569400	–0.69667400
H	–15.85079900	1.86905000	1.27396300
C	–15.25389800	1.51287700	–2.53022800
H	–13.52330600	0.35193100	–1.99608500
C	–16.35944100	2.22744600	–2.06980200
H	–17.42356500	2.90333800	–0.32437300
H	–15.08177100	1.40499900	–3.59542900
H	–17.04688900	2.68494900	–2.77159200
C	14.19760300	0.26479400	1.79565500
C	13.63563600	0.08858200	3.06731100
C	15.28278300	1.13859400	1.64670700
C	14.14438800	0.78146800	4.16189200
H	12.80133400	–0.59113000	3.19208200
C	15.79569100	1.81547600	2.74948900
H	15.72142300	1.28142500	0.66652800
C	15.22854100	1.64575100	4.01197800
H	13.69919300	0.63274200	5.13946100
H	16.63526700	2.48884900	2.61698800
H	15.62646100	2.17884200	4.86748000
C	–14.19766700	–0.26508800	1.79555400
C	–13.63564500	–0.08918900	3.06723100
C	–15.28298900	–1.13869500	1.64648300
C	–14.14447700	–0.78219300	4.16170000
H	–12.80124000	0.59037600	3.19211100
C	–15.79597200	–1.81569400	2.74915800
H	–15.72167800	–1.28129200	0.66629300
C	–15.22876600	–1.64628300	4.01166400
H	–13.69923300	–0.63370400	5.13928300
H	–16.63565600	–2.48891100	2.61655600
H	–15.62674600	–2.17946500	4.86708100
H	–3.81653500	–4.20273700	–2.25317600
H	–1.68718800	–5.42984800	1.26641600

H	3.81661600	4.20285300	–2.25304700
H	1.68722900	5.42993100	1.26653300
C	–3.59697600	–6.24728400	–0.47999100
C	3.59706500	6.24736500	–0.47984100
F	3.96801800	6.63370700	–1.72033500
F	4.73898900	6.15354000	0.25242800
F	2.87995100	7.25914500	0.05765400
F	–4.73879700	–6.15355700	0.25245300
F	–2.87976200	–7.25909900	0.05730500
F	–3.96809600	–6.63351400	–1.72046800

DCFOPV-TPA-cyclohexane

0 1

C	–0.64697700	1.21641600	0.32490200
C	0.74548400	1.19637600	0.32849300
C	1.42472400	–0.04857500	0.33585200
C	0.64697600	–1.21634000	0.32497800
C	–0.74548500	–1.19630000	0.32859100
C	–1.42472500	0.04865300	0.33587100
H	–1.14048100	2.17808600	0.30444800
H	1.14048200	–2.17801100	0.30459100
C	–2.88581500	0.09026300	0.25229200
C	–3.75998700	1.01210600	0.73333400
H	–3.32977600	–0.74474300	–0.27696400
C	2.88581100	–0.09019600	0.25226500
C	3.75997400	–1.01202300	0.73335200
H	3.32977500	0.74477600	–0.27704300
C	–3.30335500	2.13709800	1.49662800
N	–2.97560100	3.05168800	2.12373300
C	3.30332300	–2.13697600	1.49669100
N	2.97554500	–3.05153000	2.12383700
C	–5.22978300	0.92264300	0.54550400
C	–6.03049000	2.07247900	0.62298500
C	–5.86818700	–0.30138400	0.28690600
C	–7.40282600	2.00599000	0.42068900
H	–5.57446100	3.03476400	0.82448000
C	–7.23959100	–0.36505600	0.09245400
H	–5.30093200	–1.22389700	0.27445600
C	–8.04339700	0.78740000	0.14808800
H	–7.98101000	2.92176500	0.45313500
H	–7.70037300	–1.33281800	–0.06605400
C	5.22977200	–0.92258900	0.54551700
C	6.03046500	–2.07243200	0.62305000
C	5.86819300	0.30141800	0.28686400

C	7.40280200	–2.00597000	0.42075000
H	5.57442300	–3.03470300	0.82458700
C	7.23959800	0.36506400	0.09241000
H	5.30095000	1.22393700	0.27437300
C	8.04338900	–0.78740100	0.14809500
H	7.98097300	–2.92175100	0.45323500
H	7.70039200	1.33281300	–0.06614100
C	–9.50583800	0.71368000	–0.05972400
C	–10.38766800	1.55602500	0.63657500
C	–10.07024000	–0.20219400	–0.96226000
C	–11.76116300	1.48498600	0.44993500
H	–9.99835600	2.25332500	1.36962800
C	–11.44115900	–0.26989800	–1.16831000
H	–9.42477800	–0.84869900	–1.54595900
C	–12.31411900	0.57156200	–0.46121700
H	–12.41429900	2.13308600	1.02100200
H	–11.84141300	–0.97208200	–1.88904900
C	9.50583000	–0.71370800	–0.05972100
C	10.38765100	–1.55604100	0.63660500
C	10.07024300	0.20213000	–0.96228800
C	11.76114600	–1.48502500	0.44996100
H	9.99833100	–2.25331200	1.36968100
C	11.44116300	0.26981100	–1.16834000
H	9.42478900	0.84862500	–1.54600800
C	12.31411300	–0.57163700	–0.46122100
H	12.41427400	–2.13311500	1.02104900
H	11.84142500	0.97196700	–1.88910200
C	1.46719600	2.50001000	0.33588800
C	2.39277900	2.80974400	1.33979300
C	1.19504000	3.46060400	–0.64383900
C	3.02925700	4.04873600	1.35518400
H	2.59460200	2.09291800	2.12556900
C	1.83624200	4.69778900	–0.62024800
H	0.48274800	3.23905000	–1.42891900
C	2.76008100	5.00161400	0.37529200
H	3.24878400	5.96626600	0.39634800
C	–1.46719300	–2.49993400	0.33610400
C	–2.39272400	–2.80960500	1.34007700
C	–1.19508000	–3.46059300	–0.64357100
C	–3.02919500	–4.04860000	1.35558500
H	–2.59451100	–2.09272700	2.12581400
C	–1.83627500	–4.69778000	–0.61986400
H	–0.48282800	–3.23908900	–1.42870100
C	–2.76006300	–5.00154300	0.37574300

H	–3.24875900	–5.96619600	0.39688800
C	–1.56574400	–5.69080000	–1.72038500
C	1.56566000	5.69073600	–1.72082200
C	–4.05711000	–4.34365500	2.41733700
C	4.05723000	4.34385300	2.41686400
F	–1.81940600	–6.95992600	–1.33965800
F	1.81933200	6.95988800	–1.34018800
F	–2.33935500	–5.44829900	–2.81046600
F	2.33922800	5.44816800	–2.81091900
F	–0.28297200	–5.64546900	–2.14214400
F	0.28287100	5.64537200	–2.14252600
F	–4.13613500	–5.66067800	2.70123600
F	4.13629500	5.66089700	2.70065800
F	–5.29972800	–3.95665300	2.02277300
F	5.29982000	3.95679600	2.02226900
F	–3.80043700	–3.69606700	3.57256800
F	3.80059900	3.69635900	3.57215700
N	–13.71198600	0.50026400	–0.66086000
N	13.71198100	–0.50036100	–0.66086600
C	–14.50283200	1.68405300	–0.63818900
C	–15.72246100	1.70816200	0.05138800
C	–14.07775500	2.83822900	–1.31005700
C	–16.50056400	2.86259900	0.06195700
H	–16.05715000	0.82080800	0.57503800
C	–14.85393300	3.99346700	–1.28122500
H	–13.14002700	2.82570400	–1.85242600
C	–16.07063700	4.01298700	–0.59919600
H	–17.44145400	2.86494300	0.60101500
H	–14.51145600	4.87773900	–1.80724800
H	–16.67566400	4.91198200	–0.58408700
C	14.50281400	–1.68415800	–0.63815900
C	15.72244000	–1.70826100	0.05142400
C	14.07772700	–2.83834800	–1.30999700
C	16.50053000	–2.86270700	0.06202700
H	16.05713600	–0.82089600	0.57505100
C	14.85389200	–3.99359300	–1.28113000
H	13.14000200	–2.82582700	–1.85237100
C	16.07059400	–4.01310700	–0.59909600
H	17.44141900	–2.86504500	0.60109000
H	14.51140800	–4.87787500	–1.80713100
H	16.67561100	–4.91210900	–0.58395900
C	–14.34231800	–0.75683000	–0.88498300
C	–14.01123700	–1.86999400	–0.10055200
C	–15.30904700	–0.89526700	–1.88985700

C	–14.62902600	–3.09685900	–0.32654600
H	–13.27065600	–1.76956300	0.68375100
C	–15.93384200	–2.12173500	–2.09899200
H	–15.56899600	–0.03994500	–2.50183500
C	–15.59579900	–3.23049400	–1.32326900
H	–14.36291600	–3.94812300	0.29024900
H	–16.67955700	–2.21236000	–2.88102600
H	–16.07980700	–4.18532400	–1.49217200
C	14.34232700	0.75671900	–0.88502500
C	14.01125900	1.86991000	–0.10062800
C	15.30905900	0.89511600	–1.88990200
C	14.62906200	3.09676200	–0.32665700
H	13.27067500	1.76951100	0.68367800
C	15.93386900	2.12157000	–2.09907200
H	15.56900000	0.03977300	–2.50185400
C	15.59583700	3.23035600	–1.32338200
H	14.36296000	3.94804700	0.29011300
H	16.67958600	2.21216400	–2.88110800
H	16.07985600	4.18517600	–1.49231300

DCFOPV-TPA-dioxane

0 1

C	0.64877500	–1.21549800	0.32406800
C	–0.74374000	–1.19750000	0.32762100
C	–1.42451700	0.04648000	0.33527700
C	–0.64878600	1.21550000	0.32412700
C	0.74372800	1.19750000	0.32768000
C	1.42450400	–0.04647800	0.33527400
H	1.14393300	–2.17634200	0.30290300
H	–1.14394000	2.17634600	0.30299900
C	2.88573300	–0.08642500	0.25184900
C	3.76038800	–1.00474300	0.73845400
H	3.32879700	0.74607700	–0.28212500
C	–2.88574700	0.08643900	0.25183700
C	–3.76041000	1.00471500	0.73851000
H	–3.32880600	–0.74601800	–0.28221000
C	3.30397600	–2.12455700	1.50948200
N	2.97668100	–3.03445800	2.14362500
C	–3.30402000	2.12445000	1.50966700
N	–2.97675300	3.03429700	2.14390100
C	5.23020200	–0.91596800	0.55004700
C	6.03120600	–2.06513400	0.63396400
C	5.86816400	0.30674800	0.28418900
C	7.40351200	–1.99951600	0.43096300

H	5.57557200	–3.02645200	0.84090400
C	7.23954600	0.36959200	0.08895200
H	5.30063100	1.22901800	0.26626900
C	8.04367200	–0.78234300	0.15105900
H	7.98182100	–2.91500100	0.46868600
H	7.69992600	1.33652600	–0.07563700
C	–5.23022100	0.91595300	0.55007700
C	–6.03123400	2.06510400	0.63411300
C	–5.86817200	–0.30673800	0.28408100
C	–7.40353800	1.99949800	0.43109400
H	–5.57561000	3.02640300	0.84115900
C	–7.23955200	–0.36957200	0.08882900
H	–5.30063100	–1.22900000	0.26606300
C	–8.04368800	0.78235000	0.15105600
H	–7.98185400	2.91497500	0.46891200
H	–7.69992300	–1.33649200	–0.07586700
C	9.50605000	–0.70961200	–0.05791700
C	10.38826400	–1.54878900	0.64178000
C	10.06997700	0.20191000	–0.96519400
C	11.76165200	–1.47891900	0.45373700
H	9.99946600	–2.24267600	1.37832200
C	11.44079000	0.26832200	–1.17260400
H	9.42432300	0.84594500	–1.55139900
C	12.31412100	–0.57001200	–0.46223900
H	12.41498800	–2.12448700	1.02743600
H	11.84052200	0.96701300	–1.89701700
C	–9.50606500	0.70962800	–0.05793200
C	–10.38828700	1.54872900	0.64184500
C	–10.06998200	–0.20181000	–0.96530000
C	–11.76167400	1.47886500	0.45379300
H	–9.99949700	2.24254800	1.37845500
C	–11.44079300	–0.26821500	–1.17271700
H	–9.42432100	–0.84578100	–1.55156700
C	–12.31413300	0.57004200	–0.46227100
H	–12.41501700	2.12437200	1.02755300
H	–11.84051900	–0.96683900	–1.89719900
C	–1.46405900	–2.50183800	0.33503100
C	–2.38972300	–2.81197600	1.33875100
C	–1.19062600	–3.46245300	–0.64426800
C	–3.02491900	–4.05158600	1.35445500
H	–2.59240200	–2.09503200	2.12417000
C	–1.83075100	–4.70023200	–0.62034800
H	–0.47835600	–3.24040400	–1.42923100
C	–2.75461100	–5.00461700	0.37498500

H	–3.24245800	–5.96968800	0.39618500
C	1.46405600	2.50183300	0.33516100
C	2.38972500	2.81190000	1.33889900
C	1.19064300	3.46250100	–0.64409000
C	3.02494000	4.05149800	1.35467100
H	2.59239700	2.09490600	2.12427400
C	1.83078800	4.70026900	–0.62010100
H	0.47837400	3.24050300	–1.42906800
C	2.75464600	5.00458700	0.37525200
H	3.24251000	5.96965000	0.39650400
C	1.55847300	5.69342300	–1.71992700
C	–1.55842100	–5.69332700	–1.72022200
C	4.05229000	4.34763600	2.41657000
C	–4.05226600	–4.34779100	2.41633900
F	1.81443500	6.96246900	–1.34031400
F	–1.81429700	–6.96240100	–1.34064800
F	2.32874500	5.45015100	–2.81236300
F	–2.32875000	–5.45005700	–2.81261900
F	0.27445600	5.64960300	–2.13840400
F	–0.27442300	–5.64941800	–2.13874700
F	4.12787100	5.66453300	2.70261300
F	–4.12787500	–5.66471000	2.70227300
F	5.29608200	3.96479400	2.02155700
F	–5.29605200	–3.96488800	2.02136800
F	3.79770700	3.69777300	3.57107900
F	–3.79765900	–3.69803000	3.57089900
N	13.71184800	–0.50016800	–0.66342800
N	–13.71186000	0.50020300	–0.66346900
C	14.50230500	–1.68413200	–0.63571400
C	15.72230800	–1.70545900	0.05333200
C	14.07646200	–2.84127500	–1.30200700
C	16.50005800	–2.86012300	0.06885700
H	16.05760700	–0.81586900	0.57278500
C	14.85227400	–3.99666700	–1.26824500
H	13.13848000	–2.83100900	–1.84398500
C	16.06937200	–4.01344500	–0.58675500
H	17.44122600	–2.86027900	0.60743500
H	14.50920300	–4.88324200	–1.78998500
H	16.67409700	–4.91257400	–0.56779600
C	–14.50232500	1.68415900	–0.63564500
C	–15.72232600	1.70541700	0.05340800
C	–14.07649100	2.84136500	–1.30183400
C	–16.50008200	2.86007500	0.06904000
H	–16.05761800	0.81577800	0.57278200

C	–14.85231000	3.99674900	–1.26796500
H	–13.13851000	2.83115400	–1.84381600
C	–16.06940600	4.01345800	–0.58647000
H	–17.44124900	2.86017600	0.60762000
H	–14.50924500	4.88337400	–1.78962600
H	–16.67413600	4.91258200	–0.56742700
C	14.34247200	0.75557200	–0.89427400
C	14.01278800	1.87263500	–0.11478500
C	15.30811900	0.88875700	–1.90092600
C	14.63090100	3.09815200	–0.34733000
H	13.27311300	1.77635800	0.67089400
C	15.93327400	2.11393900	–2.11664900
H	15.56696900	0.03046500	–2.50920400
C	15.59662000	3.22658200	–1.34582000
H	14.36588700	3.95246700	0.26570500
H	16.67813300	2.20045800	–2.89996300
H	16.08088400	4.18036800	–1.51982600
C	–14.34247400	–0.75552200	–0.89442000
C	–14.01277100	–1.87265100	–0.11503400
C	–15.30813100	–0.88862600	–1.90107200
C	–14.63087500	–3.09815300	–0.34768200
H	–13.27308800	–1.77643800	0.67064600
C	–15.93327700	–2.11379400	–2.11689900
H	–15.56699600	–0.03028200	–2.50927100
C	–15.59660500	–3.22650300	–1.34617200
H	–14.36584600	–3.95252000	0.26527400
H	–16.67814400	–2.20025100	–2.90021100
H	–16.08086200	–4.18027900	–1.52025800

DCFOPV-TPA-toluene

0 1

C	0.65093900	–1.21447400	0.32297500
C	–0.74163100	–1.19886100	0.32652900
C	–1.42428500	0.04394600	0.33437500
C	–0.65088100	1.21442300	0.32307700
C	0.74168900	1.19881300	0.32673700
C	1.42434800	–0.04400100	0.33447000
H	1.14801700	–2.17435500	0.30116900
H	–1.14796500	2.17430400	0.30138100
C	2.88574700	–0.08194900	0.25114300
C	3.76103500	–0.99640100	0.74359500
H	3.32772100	0.74793700	–0.28782900
C	–2.88567800	0.08189400	0.25103800
C	–3.76095200	0.99634600	0.74350700

H	–3.32765300	–0.74799100	–0.28793500
C	3.30492200	–2.11075900	1.52272600
N	2.97797500	–3.01567200	2.16415000
C	–3.30480400	2.11072900	1.52258200
N	–2.97771400	3.01565700	2.16391200
C	5.23083300	–0.90826800	0.55450200
C	6.03228100	–2.05652100	0.64622900
C	5.86820300	0.31281800	0.27980600
C	7.40452900	–1.99187100	0.44237200
H	5.57714200	–3.01660200	0.85987700
C	7.23954000	0.37473500	0.08366100
H	5.30031700	1.23474400	0.25524200
C	8.04412400	–0.77645600	0.15363800
H	7.98309100	–2.90690800	0.48643100
H	7.69943100	1.34063300	–0.08820900
C	–5.23075500	0.90822700	0.55444400
C	–6.03218500	2.05649900	0.64608300
C	–5.86815300	–0.31287400	0.27987200
C	–7.40443700	1.99185300	0.44224800
H	–5.57703000	3.01659300	0.85963800
C	–7.23949200	–0.37478800	0.08375100
H	–5.30028100	–1.23481200	0.25539600
C	–8.04405900	0.77642400	0.15363000
H	–7.98297900	2.90690500	0.48623000
H	–7.69939900	–1.34069600	–0.08801600
C	9.50640200	–0.70484200	–0.05669400
C	10.38914500	–1.53964000	0.64760700
C	10.06966900	0.20097100	–0.97011100
C	11.76239400	–1.47109100	0.45795200
H	10.00097900	–2.22895900	1.38874700
C	11.44034400	0.26592500	–1.17908100
H	9.42367100	0.84151000	–1.55975600
C	12.31419300	–0.56808400	–0.46425800
H	12.41607600	–2.11314300	1.03519100
H	11.83944300	0.96003200	–1.90823500
C	–9.50634000	0.70481300	–0.05667700
C	–10.38906100	1.53973500	0.64750800
C	–10.06964000	–0.20111700	–0.96996000
C	–11.76231300	1.47118800	0.45787800
H	–10.00087600	2.22915600	1.38854200
C	–11.44031800	–0.26607200	–1.17890700
H	–9.42366600	–0.84175000	–1.55952800
C	–12.31414200	0.56805800	–0.46419300
H	–12.41597300	2.11334300	1.03502800

H	–11.83943900	–0.96027300	–1.90795900
C	–1.46027300	–2.50405200	0.33438000
C	–2.38613100	–2.81427300	1.33790600
C	–1.18520600	–3.46514300	–0.64395200
C	–3.01975600	–4.05463800	1.35450700
H	–2.58995400	–2.09684800	2.12256400
C	–1.82395800	–4.70364700	–0.61915400
H	–0.47286700	–3.24289000	–1.42879500
C	–2.74790000	–5.00827100	0.37602200
H	–3.23464300	–5.97387900	0.39782900
C	1.46029700	2.50402400	0.33484500
C	2.38592000	2.81418900	1.33860600
C	1.18536200	3.46522000	–0.64342000
C	3.01945800	4.05459600	1.35549000
H	2.58962000	2.09668900	2.12322600
C	1.82403400	4.70375900	–0.61834600
H	0.47319500	3.24301600	–1.42843400
C	2.74774800	5.00832500	0.37706000
H	3.23441700	5.97396500	0.39908800
C	1.54985600	5.69766600	–1.71691700
C	–1.54961600	–5.69744000	–1.71778800
C	4.04610900	4.35140000	2.41780600
C	–4.04666400	–4.35149400	2.41655800
F	1.80632300	6.96655800	–1.33700700
F	–1.80623700	–6.96636100	–1.33808300
F	2.31818500	5.45539100	–2.81107700
F	–2.31770500	–5.45498800	–2.81207700
F	0.26508200	5.65420300	–2.13335700
F	–0.26475400	–5.65400500	–2.13395900
F	4.11849200	5.66808400	2.70617100
F	–4.11915900	–5.66820100	2.70480000
F	5.29098200	3.97241200	2.02248700
F	–5.29143200	–3.97243600	2.02097500
F	3.79314900	3.69912100	3.57138400
F	–3.79395600	–3.69932000	3.57025000
N	13.71175900	–0.49988100	–0.66720300
N	–13.71170900	0.49985900	–0.66711300
C	14.50190500	–1.68389400	–0.63284300
C	15.72240700	–1.70135700	0.05546000
C	14.07526200	–2.84497200	–1.29176500
C	16.49986900	–2.85614000	0.07750400
H	16.05834700	–0.80875800	0.56931200
C	14.85078500	–4.00038200	–1.25150400
H	13.13692500	–2.83780500	–1.83318200

C	16.06838400	–4.01334700	–0.57076100
H	17.44141000	–2.85326400	0.61542000
H	14.50708700	–4.89002200	–1.76758400
H	16.67286600	–4.91252100	–0.54673100
C	–14.50183000	1.68389400	–0.63288500
C	–15.72231400	1.70147100	0.05544600
C	–14.07517200	2.84487900	–1.29195900
C	–16.49975000	2.85627500	0.07736100
H	–16.05826100	0.80894300	0.56941700
C	–14.85066700	4.00031300	–1.25182700
H	–13.13684500	2.83762200	–1.83339300
C	–16.06825200	4.01338900	–0.57106000
H	–17.44127900	2.85348900	0.61529800
H	–14.50696000	4.88988100	–1.76802400
H	–16.67271300	4.91258000	–0.54713000
C	14.34253400	0.75417500	–0.90665300
C	14.01412000	1.87623600	–0.13381600
C	15.30709100	0.88068900	–1.91522900
C	14.63239400	3.10007800	–0.37476000
H	13.27536100	1.78520200	0.65334900
C	15.93244600	2.10428500	–2.13937500
H	15.56495500	0.01854800	–2.51846500
C	15.59703100	3.22190100	–1.37516900
H	14.36837000	3.95831400	0.23320300
H	16.67644300	2.18559900	–2.92406300
H	16.08142700	4.17440600	–1.55571500
C	–14.34251600	–0.75420800	–0.90642300
C	–14.01411600	–1.87619500	–0.13347200
C	–15.30708900	–0.88080700	–1.91497400
C	–14.63242200	–3.10004800	–0.37427700
H	–13.27534400	–1.78509200	0.65367300
C	–15.93247400	–2.10441300	–2.13898100
H	–15.56494200	–0.01872400	–2.51829700
C	–15.59707500	–3.22195400	–1.37466000
H	–14.36840900	–3.95822600	0.23377200
H	–16.67648400	–2.18579400	–2.92365100
H	–16.08149500	–4.17446800	–1.55509900

DCFOPV-TPA-chloroform

0 1

C	–0.66733500	1.20621200	0.34687200
C	0.72547100	1.20918200	0.35173300
C	1.42304600	–0.02449300	0.35673600
C	0.66734300	–1.20601400	0.34692500

C	–0.72546300	–1.20898400	0.35179500
C	–1.42303900	0.02469100	0.35674600
H	–1.17885700	2.15863500	0.32161500
H	1.17886800	–2.15843800	0.32171300
C	–2.88489500	0.04605200	0.26426700
C	–3.77008500	0.93396000	0.78557200
H	–3.31267900	–0.76814300	–0.30920200
C	2.88490100	–0.04586900	0.26425100
C	3.77007300	–0.93377500	0.78558900
H	3.31269200	0.76829300	–0.30926000
C	–3.32141000	2.01047500	1.62071000
N	–3.00121400	2.88051200	2.31192200
C	3.32136000	–2.01024900	1.62076000
N	3.00111900	–2.88024900	2.31199800
C	–5.23829100	0.85198300	0.57982300
C	–6.04656300	1.98669000	0.74924300
C	–5.86792100	–0.34806600	0.20968000
C	–7.41749000	1.93152000	0.53214500
H	–5.59869000	2.93130200	1.03481600
C	–7.23767400	–0.40097500	–0.00057100
H	–5.29631200	–1.26309800	0.11583800
C	–8.04900800	0.73803600	0.14964400
H	–8.00000900	2.83859800	0.64069200
H	–7.69039600	–1.35305100	–0.25072400
C	5.23828100	–0.85185700	0.57982900
C	6.04651800	–1.98657700	0.74933300
C	5.86795000	0.34814300	0.20959200
C	7.41744600	–1.93146900	0.53222900
H	5.59861400	–2.93115300	1.03497800
C	7.23770500	0.40099200	–0.00066400
H	5.29637300	1.26318700	0.11567600
C	8.04900200	–0.73803400	0.14963700
H	7.99993500	–2.83855800	0.64084300
H	7.69045700	1.35303300	–0.25089200
C	–9.50963600	0.67551400	–0.07658900
C	–10.40137100	1.45755900	0.67569500
C	–10.06206800	–0.16852200	–1.05388700
C	–11.77301400	1.39662100	0.47092900
H	–10.02238900	2.09779200	1.46403200
C	–11.43103000	–0.22436900	–1.27739300
H	–9.40938500	–0.76604300	–1.68009200
C	–12.31391300	0.55583200	–0.51445100
H	–12.43333200	1.99589200	1.08543400
H	–11.82099400	–0.86916400	–2.05517200

C	9.50963300	–0.67557800	–0.07660100
C	10.40134100	–1.45759900	0.67574000
C	10.06209300	0.16836900	–1.05396000
C	11.77298600	–1.39672200	0.47097000
H	10.02233700	–2.09776200	1.46412300
C	11.43105700	0.22415300	–1.27747000
H	9.40943000	0.76586600	–1.68020800
C	12.31391300	–0.55602300	–0.51447000
H	12.43328300	–1.99597100	1.08551800
H	11.82104400	0.86887900	–2.05529500
C	1.43039100	2.52132700	0.36768500
C	2.35433000	2.83065400	1.37325000
C	1.14504500	3.48818300	–0.60185300
C	2.97572900	4.07686100	1.40104600
H	2.56573400	2.10764100	2.15055900
C	1.77285400	4.73215400	–0.56590500
H	0.43446000	3.26585500	–1.38824700
C	2.69471300	5.03677400	0.43130300
H	3.17296000	6.00633500	0.46095000
C	–1.43038200	–2.52112900	0.36781700
C	–2.35430600	–2.83041100	1.37341000
C	–1.14505100	–3.48802700	–0.60168300
C	–2.97570600	–4.07661600	1.40126900
H	–2.56569700	–2.10736400	2.15069000
C	–1.77286000	–4.73199700	–0.56567100
H	–0.43447900	–3.26573400	–1.38809800
C	–2.69470500	–5.03657200	0.43156500
H	–3.17295200	–6.00613200	0.46126100
C	–1.48703600	–5.73341300	–1.65361400
C	1.48701300	5.73352200	–1.65388800
C	–3.99653500	–4.37526500	2.46799700
C	3.99657400	4.37555700	2.46774600
F	–1.74190500	–7.00105600	–1.26691200
F	1.74188000	7.00118300	–1.26724200
F	–2.24651200	–5.50393100	–2.75748600
F	2.24648000	5.50399700	–2.75775700
F	–0.19873600	–5.69027900	–2.06101300
F	0.19871000	5.69036400	–2.06127300
F	–4.05171500	–5.69032600	2.77106600
F	4.05175800	5.69063100	2.77075600
F	–5.24816000	–4.01726600	2.07444400
F	5.24819300	4.01754100	2.07419200
F	–3.74981800	–3.70910600	3.61581700
F	3.74987300	3.70945000	3.61559900

N	–13.70982600	0.49579100	–0.73221700
N	13.70982900	–0.49604500	–0.73224100
C	–14.50487000	1.67242100	–0.62764100
C	–15.72977100	1.64177500	0.05264000
C	–14.07855300	2.87472500	–1.20845200
C	–16.51208500	2.79018100	0.14309900
H	–16.06557200	0.71754800	0.50728700
C	–14.85891700	4.02271800	–1.09990700
H	–13.13706600	2.90581200	–1.74352900
C	–16.08103900	3.98803300	–0.42754500
H	–17.45686900	2.74972200	0.67375900
H	–14.51537000	4.94454500	–1.55609800
H	–16.68920000	4.88172000	–0.35027900
C	14.50483100	–1.67269500	–0.62758900
C	15.72973600	–1.64204800	0.05268600
C	14.07847100	–2.87502200	–1.20832100
C	16.51201000	–2.79047500	0.14321600
H	16.06557000	–0.71780300	0.50727200
C	14.85879500	–4.02303500	–1.09970500
H	13.13698100	–2.90611100	–1.74339400
C	16.08092100	–3.98835000	–0.42734800
H	17.45679700	–2.75001600	0.67387200
H	14.51521400	–4.94488000	–1.55583500
H	16.68905100	–4.88205300	–0.35002600
C	–14.33350000	–0.74268900	–1.05621000
C	–14.00540400	–1.91085700	–0.35461600
C	–15.29041400	–0.80816900	–2.07800700
C	–14.61617900	–3.11949700	–0.67829200
H	–13.27316800	–1.86792500	0.44265900
C	–15.90849600	–2.01760900	–2.38513300
H	–15.54803700	0.08947100	–2.62716400
C	–15.57308200	–3.18092400	–1.69192800
H	–14.35231800	–4.01383800	–0.12474800
H	–16.64641000	–2.05137900	–3.17899400
H	–16.05158700	–4.12190900	–1.93709300
C	14.33354400	0.74239300	–1.05631300
C	14.00548700	1.91061800	–0.35479600
C	15.29046200	0.80777500	–2.07811400
C	14.61630200	3.11921600	–0.67854800
H	13.27324900	1.86776100	0.44248200
C	15.90858400	2.01717500	–2.38531700
H	15.54805600	–0.08990900	–2.62721200
C	15.57320800	3.18054600	–1.69218700
H	14.35247100	4.01360200	–0.12506200

H	16.64650000	2.05086900	–3.17918000
H	16.05174400	4.12149900	–1.93741400

DCFOPV-TPA-thf

0 1

C	–0.67334000	1.20256800	0.36083200
C	0.71963600	1.21324200	0.36503100
C	1.42249600	–0.01706900	0.36992600
C	0.67334400	–1.20248300	0.36089900
C	–0.71963200	–1.21315700	0.36509900
C	–1.42249200	0.01715500	0.36992600
H	–1.19030400	2.15206300	0.33433700
H	1.19030800	–2.15197900	0.33445700
C	–2.88461800	0.03409400	0.27369200
C	–3.77196800	0.91246900	0.80664100
H	–3.30827800	–0.77195900	–0.31432300
C	2.88462100	–0.03401600	0.27369200
C	3.77196800	–0.91236200	0.80669400
H	3.30828300	0.77199700	–0.31437600
C	–3.32537900	1.97358600	1.66233300
N	–3.00811100	2.82930600	2.37257200
C	3.32537200	–1.97341900	1.66245800
N	3.00809700	–2.82908700	2.37275700
C	–5.23980700	0.83399500	0.59538400
C	–6.04871800	1.96626400	0.77831700
C	–5.86794900	–0.36052400	0.20539300
C	–7.41903100	1.91434100	0.55583100
H	–5.60200200	2.90692500	1.07839100
C	–7.23706800	–0.41033400	–0.01042600
H	–5.29575100	–1.27368300	0.09908100
C	–8.04902400	0.72639800	0.15375600
H	–8.00194800	2.81974300	0.67579900
H	–7.68851200	–1.35843900	–0.27740400
C	5.23980700	–0.83391700	0.59542400
C	6.04870800	–1.96617800	0.77844300
C	5.86795900	0.36056600	0.20533600
C	7.41902000	–1.91428700	0.55594600
H	5.60198400	–2.90681200	1.07859200
C	7.23707700	0.41034500	–0.01049400
H	5.29576900	1.27372200	0.09895200
C	8.04902300	–0.72638200	0.15377500
H	8.00192900	–2.81968500	0.67598300
H	7.68852900	1.35842500	–0.27754900
C	–9.50895000	0.66686300	–0.07872800

C	–10.40365400	1.43976300	0.67966100
C	–10.05764600	–0.16551700	–1.06822900
C	–11.77462700	1.38089100	0.46922200
H	–10.02779300	2.07129500	1.47647700
C	–11.42586800	–0.21916400	–1.29710400
H	–9.40265900	–0.75564900	–1.69900000
C	–12.31185700	0.55152600	–0.52792100
H	–12.43708600	1.97280400	1.08853500
H	–11.81258100	–0.85480900	–2.08398900
C	9.50894900	–0.66688100	–0.07872000
C	10.40364600	–1.43972900	0.67972800
C	10.05764900	0.16541200	–1.06829000
C	11.77462000	–1.38089100	0.46927800
H	10.02778200	–2.07119200	1.47659700
C	11.42587200	0.21902500	–1.29717600
H	9.40266700	0.75550200	–1.69910600
C	12.31185500	–0.55161400	–0.52793600
H	12.43707400	–1.97276100	1.08863700
H	11.81258800	0.85460100	–2.08411500
C	1.41780300	2.52866000	0.37933600
C	2.34947500	2.83982700	1.37686300
C	1.11807400	3.49743900	–0.58442300
C	2.96431000	4.08957900	1.40302500
H	2.57202400	2.11567600	2.14998200
C	1.73939600	4.74443000	–0.55002400
H	0.40166500	3.27369100	–1.36508700
C	2.66935800	5.05099700	0.43939900
H	3.14289600	6.02286800	0.46762800
C	–1.41779900	–2.52857500	0.37947100
C	–2.34946500	–2.83969600	1.37701800
C	–1.11807400	–3.49739900	–0.58424400
C	–2.96430100	–4.08944700	1.40324000
H	–2.57201100	–2.11550900	2.15010400
C	–1.73939600	–4.74438800	–0.54978500
H	–0.40166900	–3.27368700	–1.36492100
C	–2.66935400	–5.05090900	0.43965700
H	–3.14289200	–6.02277800	0.46793300
C	–1.43861300	–5.74856900	–1.63110100
C	1.43860600	5.74856100	–1.63138500
C	–3.99207400	–4.38840200	2.46308000
C	3.99208900	4.38858200	2.46284600
F	–1.67941700	–7.01719200	–1.23771900
F	1.67941800	7.01720100	–1.23806500
F	–2.19737000	–5.53573300	–2.73884100

F	2.19735100	5.53567000	–2.73912300
F	–0.14984500	–5.69354100	–2.03557500
F	0.14983300	5.69351800	–2.03584400
F	–4.06314400	–5.70554900	2.75321300
F	4.06317400	5.70574400	2.75290800
F	–5.23876900	–4.01234700	2.07091700
F	5.23877900	4.01249100	2.07070000
F	–3.74172100	–3.73709100	3.61927700
F	3.74173200	3.73733600	3.61907800
N	–13.70692500	0.49314800	–0.75114200
N	13.70692200	–0.49327200	–0.75116800
C	–14.50358100	1.66768700	–0.63525900
C	–15.73023100	1.62793700	0.04156400
C	–14.07721200	2.87698100	–1.20152500
C	–16.51434100	2.77435500	0.14275300
H	–16.06603300	0.69842800	0.48534000
C	–14.85934500	4.02282400	–1.08233400
H	–13.13450300	2.91525300	–1.73398600
C	–16.08328900	3.97911500	–0.41350200
H	–17.46041400	2.72685000	0.67052500
H	–14.51577900	4.95006500	–1.52738900
H	–16.69280400	4.87112500	–0.32791500
C	14.50356400	–1.66781200	–0.63519500
C	15.73021800	–1.62802300	0.04161900
C	14.07717600	–2.87714600	–1.20136300
C	16.51431300	–2.77444500	0.14289500
H	16.06603400	–0.69848400	0.48531900
C	14.85929500	–4.02298900	–1.08208500
H	13.13446400	–2.91544700	–1.73381600
C	16.08324300	–3.97924300	–0.41326200
H	17.46038900	–2.72690900	0.67065900
H	14.51571500	–4.95026100	–1.52706400
H	16.69274700	–4.87125300	–0.32760700
C	–14.32860800	–0.74191900	–1.09204100
C	–14.00417100	–1.91754800	–0.40117100
C	–15.27974100	–0.79647800	–2.11994600
C	–14.61285100	–3.12284400	–0.74123700
H	–13.27660300	–1.88310200	0.40078200
C	–15.89578600	–2.00274600	–2.44371800
H	–15.53433100	0.10688400	–2.66110600
C	–15.56398500	–3.17348900	–1.76107600
H	–14.35191500	–4.02303700	–0.19585600
H	–16.62907600	–2.02815600	–3.24215900
H	–16.04079100	–4.11187800	–2.01919600

C	14.32861900	0.74176000	–1.09217000
C	14.00420200	1.91744900	–0.40139400
C	15.27974800	0.79622300	–2.12008400
C	14.61289500	3.12270900	–0.74156000
H	13.27663800	1.88307600	0.40056600
C	15.89580600	2.00245700	–2.44395800
H	15.53432300	–0.10718600	–2.66117300
C	15.56402400	3.17326000	–1.76140900
H	14.35197400	4.02294900	–0.19625100
H	16.62909300	2.02779400	–3.24240500
H	16.04084000	4.11162200	–2.01960800

DCFOPV-TPA-DCM

0 1

C	0.67404700	–1.20244700	0.35884600
C	–0.71882800	–1.21359200	0.36355800
C	–1.42239900	0.01638400	0.36822400
C	–0.67404800	1.20235800	0.35889300
C	0.71882700	1.21350300	0.36361300
C	1.42239800	–0.01647300	0.36823100
H	1.19160400	–2.15163600	0.33117600
H	–1.19160500	2.15154900	0.33125900
C	2.88421000	–0.03164600	0.27226500
C	3.77267400	–0.90940500	0.80455900
H	3.30676200	0.77558100	–0.31491400
C	–2.88421000	0.03156500	0.27225000
C	–3.77267200	0.90929500	0.80459500
H	–3.30676200	–0.77562500	–0.31498200
C	3.32704300	–1.96891000	1.66267200
N	3.01012200	–2.82310900	2.37496500
C	–3.32703500	1.96873600	1.66278400
N	–3.01010900	2.82288100	2.37514000
C	5.24032500	–0.83119400	0.59184600
C	6.05025400	–1.96114200	0.78388800
C	5.86751200	0.36043400	0.19149100
C	7.42066000	–1.90992900	0.56127500
H	5.60464900	–2.89999400	1.09119000
C	7.23664000	0.40960500	–0.02453300
H	5.29473300	1.27209200	0.07610100
C	8.04978800	–0.72482000	0.14960600
H	8.00386800	–2.81403300	0.68903100
H	7.68671700	1.35586700	–0.30012300
C	–5.24032300	0.83111500	0.59186800
C	–6.05024400	1.96104900	0.78402000

C	–5.86751800	–0.36046900	0.19139000
C	–7.42065000	1.90986900	0.56139800
H	–5.60463300	2.89986700	1.09141700
C	–7.23664600	–0.40960800	–0.02464300
H	–5.29474500	–1.27211900	0.07590600
C	–8.04978600	0.72480600	0.14960800
H	–8.00385100	2.81396400	0.68924200
H	–7.68672800	–1.35583800	–0.30033000
C	9.50990900	–0.66582400	–0.08276500
C	10.40469600	–1.43418300	0.68019400
C	10.05862300	0.16142200	–1.07662000
C	11.77578300	–1.37576200	0.46993100
H	10.02912200	–2.06167200	1.48027600
C	11.42697400	0.21432400	–1.30525800
H	9.40392200	0.74801200	–1.71090400
C	12.31288800	–0.55172500	–0.53153800
H	12.43820800	–1.96393400	1.09285200
H	11.81363000	0.84581500	–2.09552200
C	–9.50990700	0.66584400	–0.08276900
C	–10.40468700	1.43413900	0.68026300
C	–10.05862800	–0.16130000	–1.07670500
C	–11.77577500	1.37575200	0.46999300
H	–10.02910800	2.06154700	1.48040600
C	–11.42697900	–0.21416700	–1.30534900
H	–9.40393300	–0.74783600	–1.71104500
C	–12.31288700	0.55181700	–0.53155600
H	–12.43819400	1.96387000	1.09297000
H	–11.81364100	–0.84557900	–2.09567400
C	–1.41708800	–2.52903900	0.38000800
C	–2.34243100	–2.84056600	1.38335400
C	–1.12362900	–3.49671000	–0.58655300
C	–2.95713300	–4.09015700	1.41252900
H	–2.56029100	–2.11656600	2.15788300
C	–1.74552300	–4.74352000	–0.54928100
H	–0.41208300	–3.27220600	–1.37140100
C	–2.66885900	–5.05086400	0.44594300
H	–3.14279100	–6.02244800	0.47600800
C	1.41708600	2.52895000	0.38012200
C	2.34241300	2.84044200	1.38349400
C	1.12364100	3.49665500	–0.58640900
C	2.95711400	4.09003300	1.41272200
H	2.56026200	2.11641500	2.15800100
C	1.74553400	4.74346400	–0.54908400
H	0.41210700	3.27217900	–1.37127600

C	2.66885400	5.05077400	0.44616600
H	3.14278500	6.02235700	0.47627200
C	1.45125200	5.74634000	–1.63291900
C	–1.45122500	–5.74635700	–1.63314700
C	3.97653200	4.39169900	2.47932900
C	–3.97656900	–4.39186000	2.47910900
F	1.70080400	7.01477000	–1.24363600
F	–1.70077700	–7.01480100	–1.24391000
F	2.20757800	5.52520600	–2.74096000
F	–2.20753800	–5.52518900	–2.74119000
F	0.16149200	5.69887100	–2.03650500
F	–0.16146000	–5.69887000	–2.03671600
F	4.03066200	5.70760300	2.78060400
F	–4.03071200	–5.70777500	2.78033100
F	5.22982400	4.03407300	2.09013200
F	–5.22985300	–4.03420900	2.08990800
F	3.72889700	3.72813300	3.62926900
F	–3.72894500	–3.72834000	3.62907900
N	13.70821200	–0.49386600	–0.75454500
N	–13.70821100	0.49399400	–0.75457100
C	14.50466800	–1.66805500	–0.63518900
C	15.73235000	–1.62602900	0.03967600
C	14.07708500	–2.87955900	–1.19583200
C	16.51624800	–2.77231400	0.14444400
H	16.06924400	–0.69498700	0.47938400
C	14.85900600	–4.02521400	–1.07305500
H	13.13365800	–2.91993600	–1.72684700
C	16.08398100	–3.97925500	–0.40620400
H	17.46309100	–2.72292300	0.67064200
H	14.51439600	–4.95414000	–1.51374700
H	16.69331200	–4.87111700	–0.31785800
C	–14.50465400	1.66818100	–0.63510800
C	–15.73233800	1.62610600	0.03975100
C	–14.07705600	2.87973200	–1.19563700
C	–16.51622300	2.77239100	0.14462300
H	–16.06924300	0.69502800	0.47937200
C	–14.85896300	4.02538500	–1.07275600
H	–13.13362600	2.92014700	–1.72664600
C	–16.08394000	3.97937800	–0.40591200
H	–17.46306700	2.72296300	0.67081400
H	–14.51434100	4.95434800	–1.51336200
H	–16.69326100	4.87123900	–0.31748500
C	14.32940200	0.74041100	–1.09891300
C	14.00366600	1.91802300	–0.41204100

C	15.28109800	0.79242300	–2.12643200
C	14.61170700	3.12271100	–0.75555200
H	13.27558600	1.88582200	0.38954200
C	15.89655600	1.99810600	–2.45365200
H	15.53663800	–0.11230200	–2.66485900
C	15.56352500	3.17081600	–1.77492100
H	14.34969400	4.02445100	–0.21327100
H	16.63029200	2.02146100	–3.25173400
H	16.03985100	4.10871400	–2.03568200
C	–14.32941400	–0.74024300	–1.09905500
C	–14.00369100	–1.91792300	–0.41229400
C	–15.28110900	–0.79214900	–2.12658100
C	–14.61174400	–3.12257300	–0.75591900
H	–13.27561200	–1.88580600	0.38929300
C	–15.89658000	–1.99779500	–2.45391400
H	–15.53664000	0.11262900	–2.66492300
C	–15.56356200	–3.17057200	–1.77529300
H	–14.34974200	–4.02436700	–0.21372200
H	–16.63031500	–2.02106800	–3.25200000
H	–16.03989800	–4.10844100	–2.03614300

NMR Spectrum

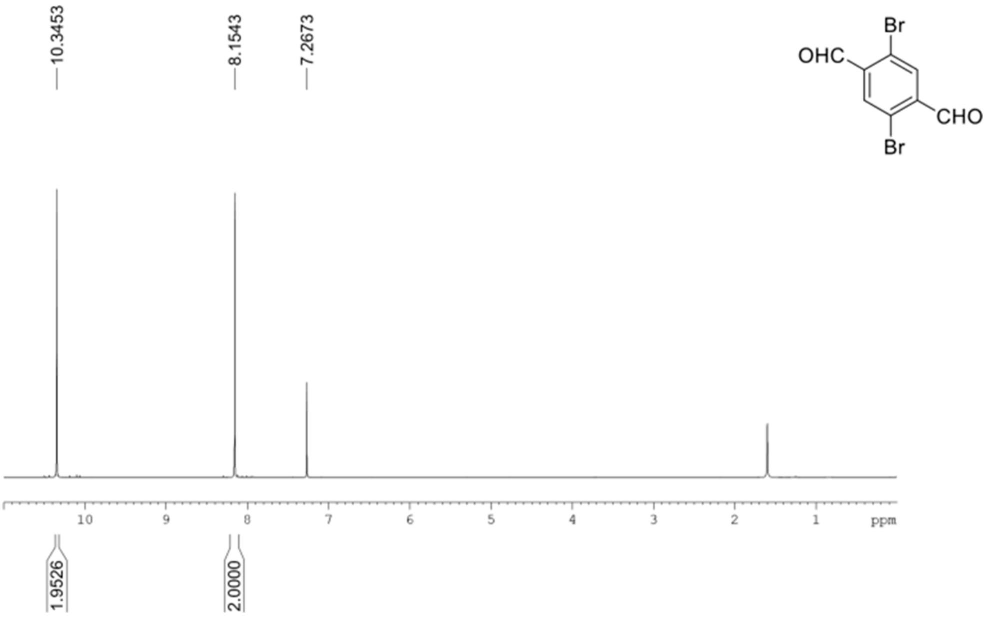

Figure S1. ^{1}H NMR spectrum (600 MHz) of compound 1 in $CDCl_3$

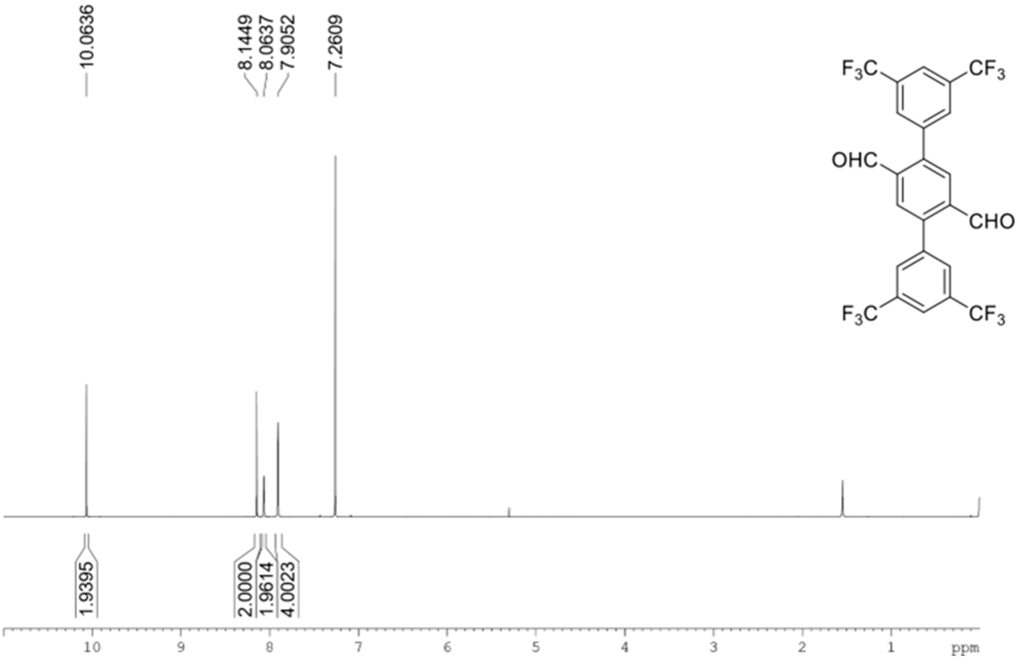

Figure S2. ^{1}H NMR spectrum (600 MHz) of compound 2a in $CDCl_3$

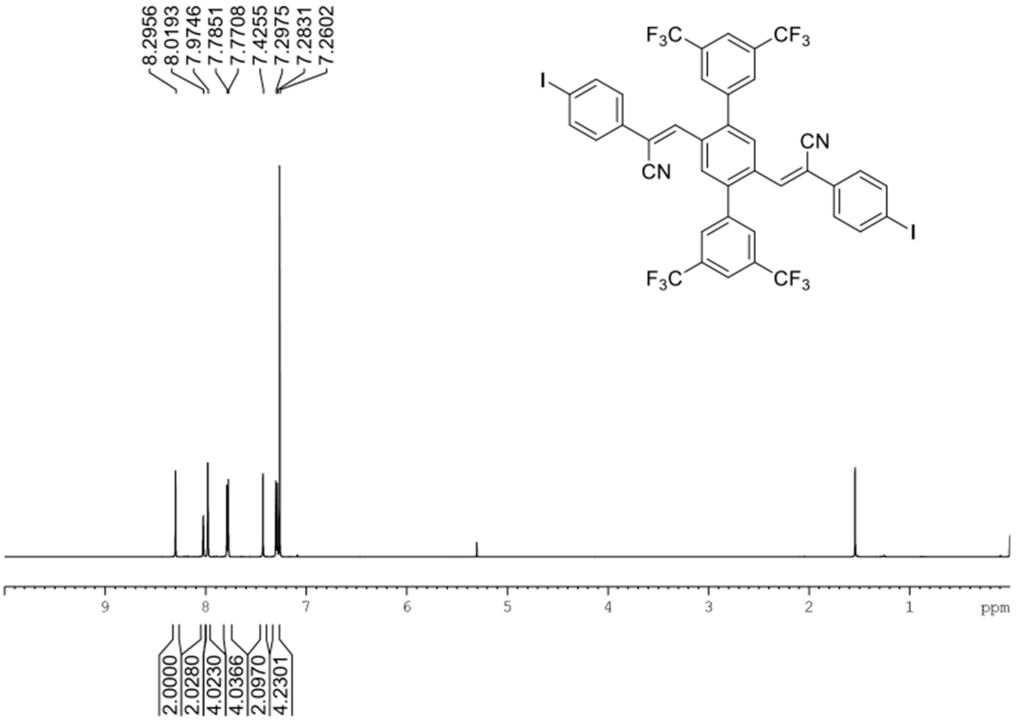

Figure S3. ^{1}H NMR spectrum (600 MHz) of compound 3a in $CDCl_3$

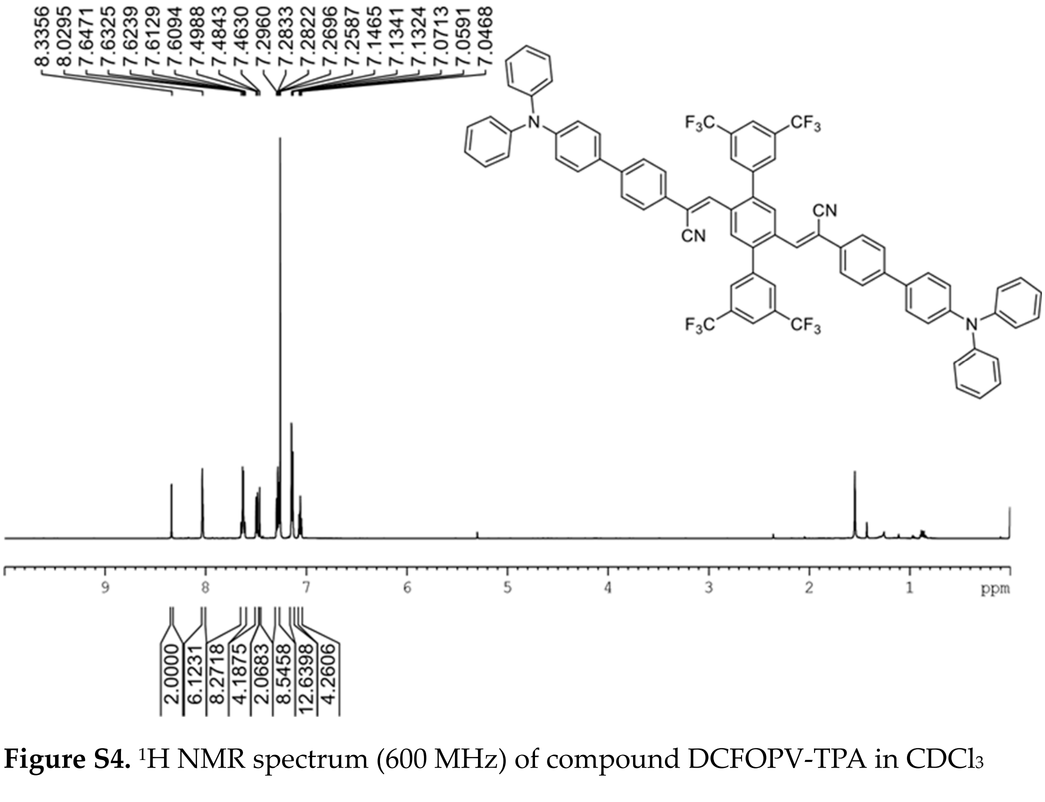

Figure S4. ^{1}H NMR spectrum (600 MHz) of compound DCFOPV-TPA in $CDCl_3$

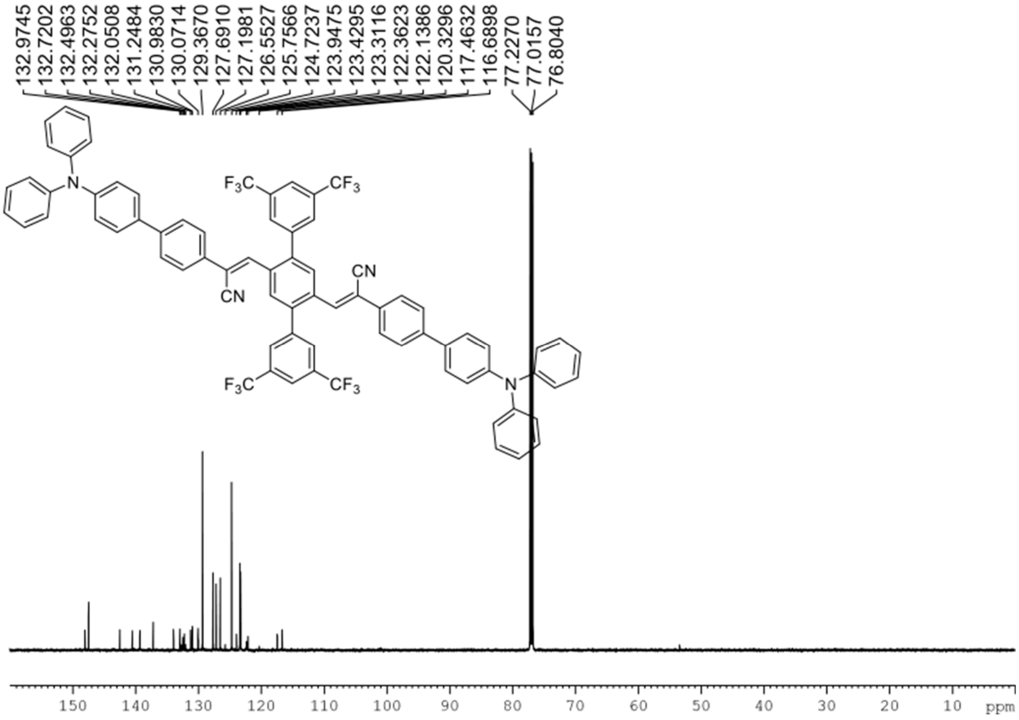

Figure S5. ^{13}C NMR spectrum (151MHz) of compound DCFOPV-TPA in $CDCl_3$

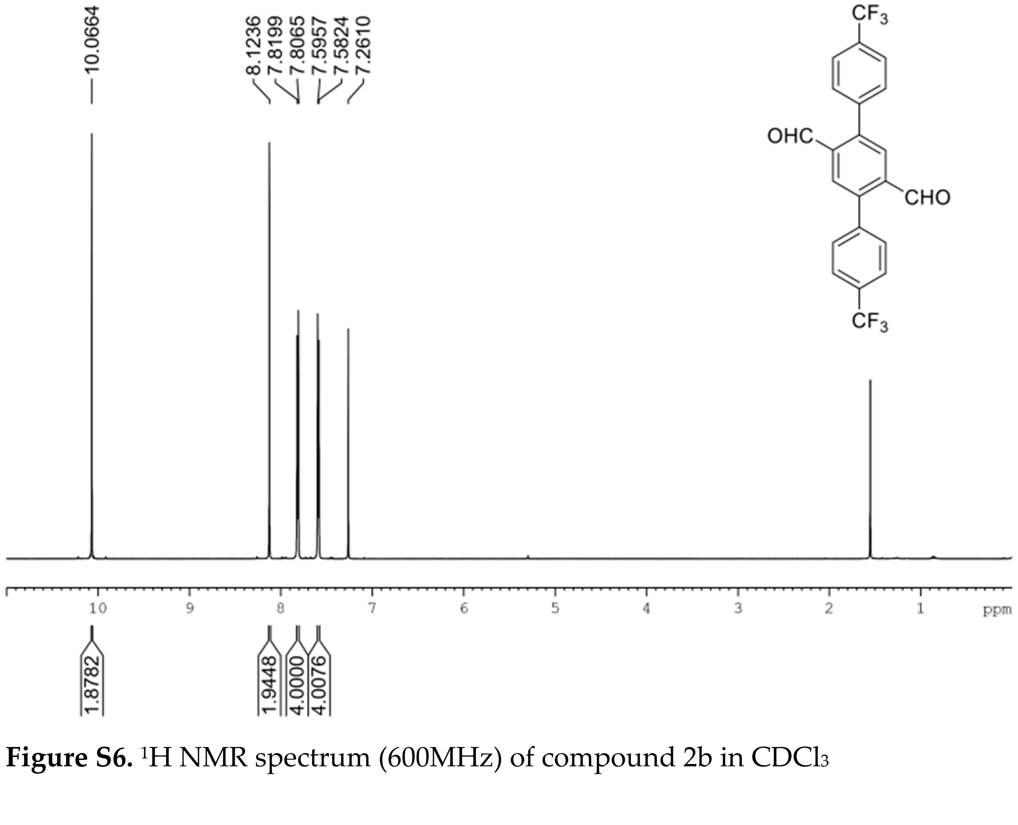

Figure S6. ^1H NMR spectrum (600MHz) of compound 2b in $CDCl_3$

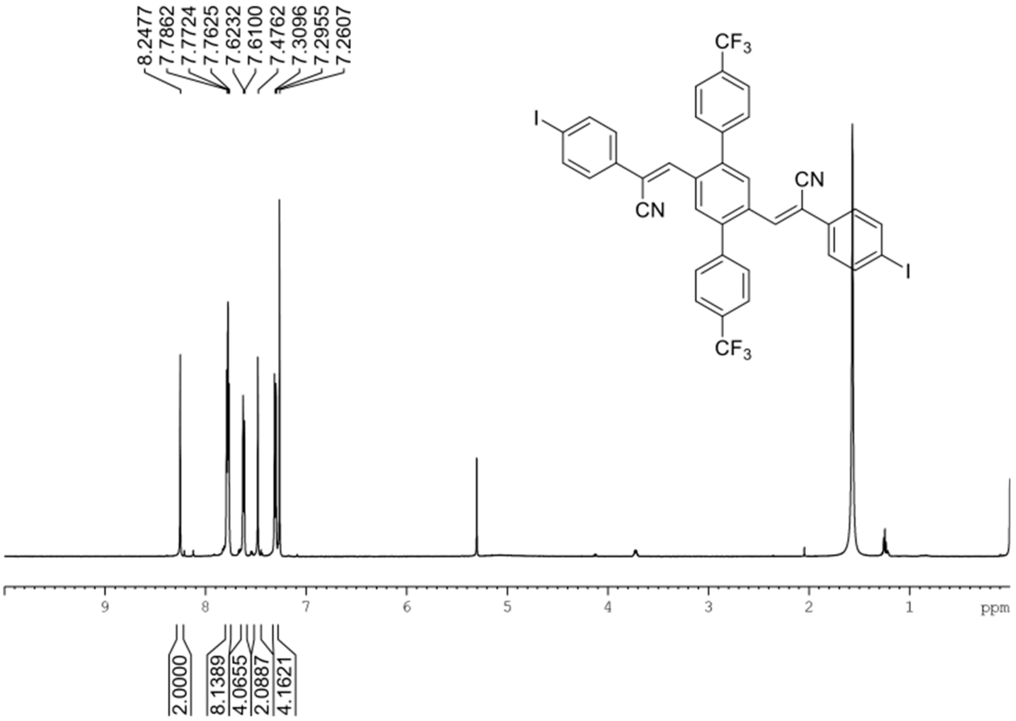

Figure S7. ¹H NMR spectrum (600MHz) of compound 3b in $CDCl_3$

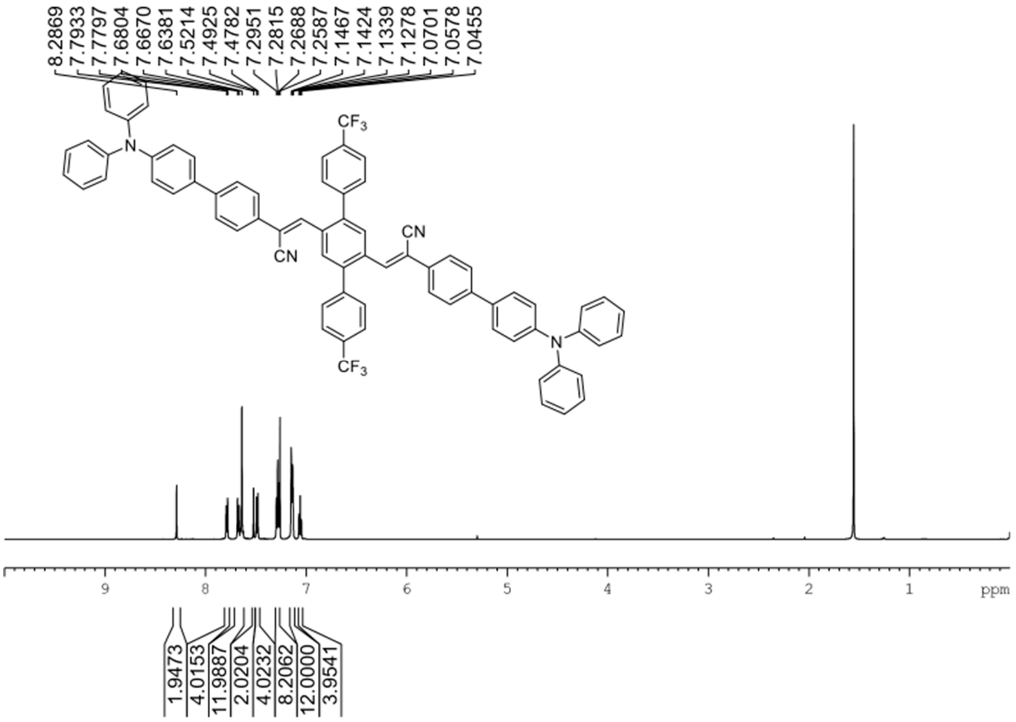

Figure S8. ¹H NMR spectrum (600MHz) of compound SCFOPV-TPA in $CDCl_3$

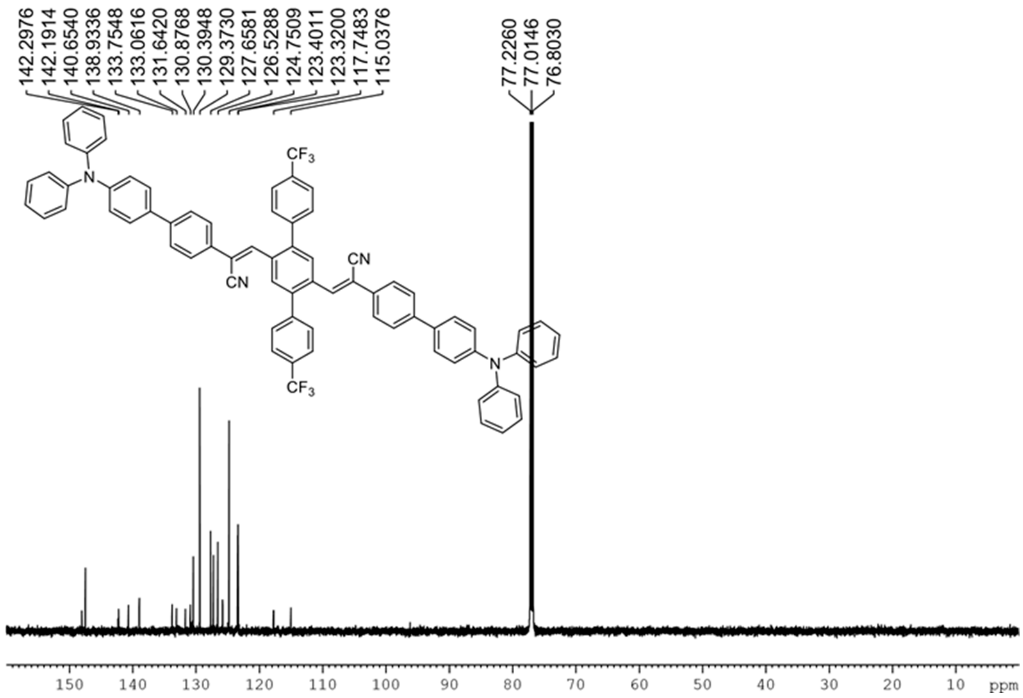

Figure S9. ^{13}C NMR spectrum (151MHz) of compound SCFOPV-TPA in $CDCl_3$

HRMS Spectra

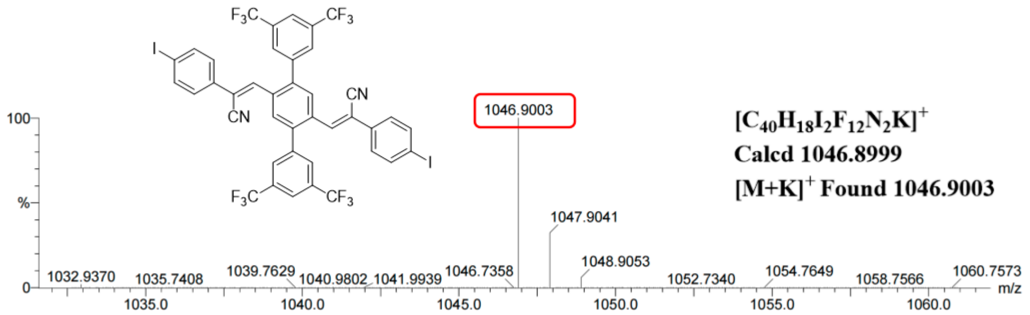

Figure S10. HRMS spectrum of 3a

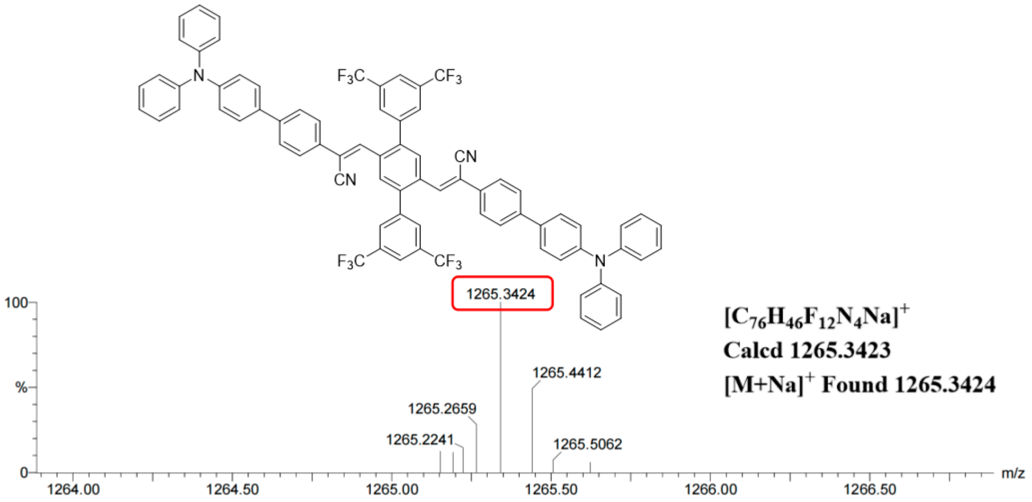

Figure S11. HRMS spectrum of DCFOPV-TPA

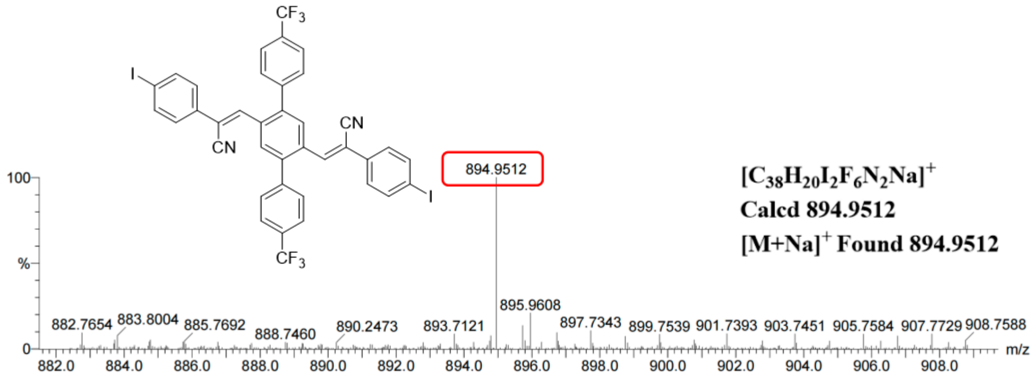

Figure S12. HRMS spectrum of 3b

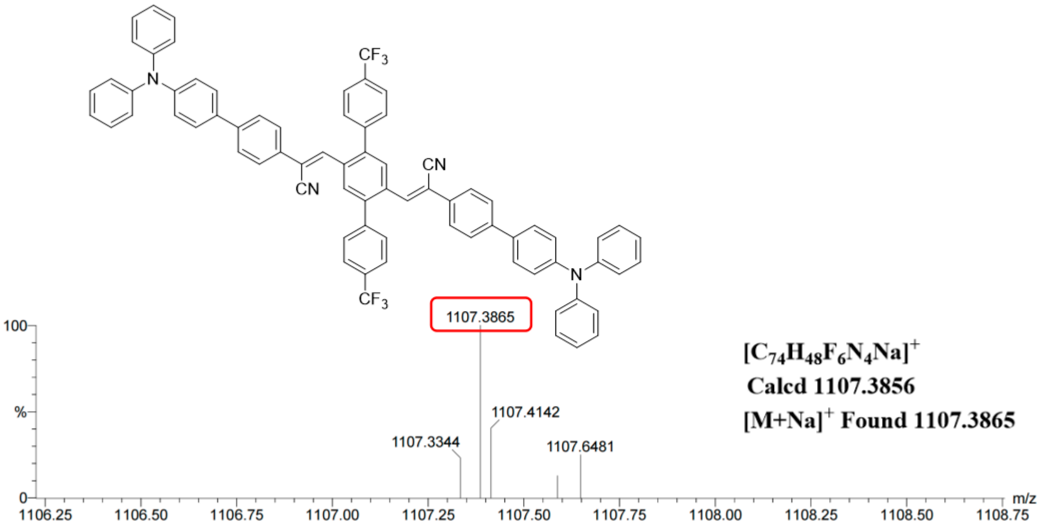

Figure S13. HRMS spectrum of SCFOPV-TPA
